# Quantum process tomography with unsupervised learning and tensor networks

**DOI:** 10.1038/s41467-023-38332-9

**Published:** 2023-05-19

**Authors:** Giacomo Torlai, Christopher J. Wood, Atithi Acharya, Giuseppe Carleo, Juan Carrasquilla, Leandro Aolita

**Affiliations:** 1 AWS Center for Quantum Computing, Pasadena, CA USA; 2 Center for Computational Quantum Physics, Flatiron Institute, New York, NY 10010 USA; 3 IBM T.J. Watson Research Center, Yorktown Heights, NY 10598 USA; 4 Physics and Astronomy Department, Rutgers University, Piscataway, NJ 08854 USA; 5 Institute of Physics, École Polytechnique Fédérale de Lausanne, CH-1015 Lausanne, Switzerland; 6 Vector Institute, MaRS Centre, Toronto, Ontario M5G 1M1 Canada; 7 Quantum Research Centre, Technology Innovation Institute, Abu Dhabi, UAE; 8 Instituto de Física, Federal University of Rio de Janeiro, 21941-972, P. O. Box 68528, Rio de Janeiro, Brazil

**Keywords:** Quantum information, Computational science

## Abstract

The impressive pace of advance of quantum technology calls for robust and scalable techniques for the characterization and validation of quantum hardware. Quantum process tomography, the reconstruction of an unknown quantum channel from measurement data, remains the quintessential primitive to completely characterize quantum devices. However, due to the exponential scaling of the required data and classical post-processing, its range of applicability is typically restricted to one- and two-qubit gates. Here, we present a technique for performing quantum process tomography that addresses these issues by combining a tensor network representation of the channel with a data-driven optimization inspired by unsupervised machine learning. We demonstrate our technique through synthetically generated data for ideal one- and two-dimensional random quantum circuits of up to 10 qubits, and a noisy 5-qubit circuit, reaching process fidelities above 0.99 using several orders of magnitude fewer (single-qubit) measurement shots than traditional tomographic techniques. Our results go far beyond state-of-the-art, providing a practical and timely tool for benchmarking quantum circuits in current and near-term quantum computers.

## Abstract (web summary)

In quantum technologies, scalable ways to characterise errors in quantum hardware are highly needed. Here, the authors propose an approximate version of quantum process tomography based on tensor network representations of the processes and data-driven optimisation.

## Introduction

Digital quantum computers and analog quantum simulators are entering regimes outside the reach of classical computing hardware^1^. Coherent manipulation of complex quantum states with dozens of qubits have been realized across several platforms, including trapped ions^2,3^, Rydberg atom arrays^4^, cold atoms in optical lattices^5^, and super-conducting qubit circuits^6,7^. In particular, programable 2D quantum circuits with between 53^6^ and 65^7^ qubits, and tens of gate layers, have been run with high fidelity in the latter platforms. Over the next few years, it is expected that quantum devices will attain hundreds of qubits, unlocking a variety of quantum computing applications with far-reaching scientific and technological ramifications.

As the size and complexity of quantum hardware continues to grow, techniques capable of characterizing complex multi-qubit error processes are essential for developing error mitigation for near-term applications^8–11^. Recent efforts have focused on generalizations of randomized benchmarking^12^ to recover partial information about the strength and locality of correlated errors in multi-qubit devices^13–15^. However, these approaches capture only averaged features (their so-called Pauli projections^14^) of the noise channel, leaving aside, e.g., all non-unital channels, including the important amplitude damping noise. Other approaches exist for the validation of average fidelities of a prepared quantum state through a reduced set of measurements^16–20^, but only provide limited information about the nature of the noise in the preparation circuit. None of the above methods tackles the characterization of arbitrary quantum processes.

Quantum process tomography (QPT)^21,22^, a procedure that reconstructs an unknown quantum process from measurement data, is a fundamental tool for diagnostic and full characterization of quantum gates and circuits. A direct approach to QPT relies on a informationally-complete (IC) set of measurement settings, which inevitably leads to an algorithmic complexity—in terms of number of measurements and classical post-processing—that scales exponentially with the number of qubits. Due to these limitations, QPT has only been experimentally implemented on up to 3 qubits^23–30^.

In most practical scenarios, however, a process to be characterized in a quantum computer typically contains structures that may facilitate its reconstruction. The origin of these structures can be traced back to, e.g., the limited availability and degree of locality of the Hamiltonians used to implement the unitary set of operations in a quantum computer, as well as the nature and strength of the inherent noise of the device, which is often local and exhibits weak correlations among the different qubits. While manipulating and reconstructing a fully generic process requires exponential classical resources^28^, these observations suggest that it may be possible to accurately describe relevant quantum channels implemented in real devices by means of classical resources with only polynomial overhead. In fact, similar insights have been leveraged successfully in quantum state tomography, the data-driven reconstruction of a quantum state. Notable examples include matrix product state (MPS) tomography^31–33^, exploiting low-entanglement representations of quantum states, and compressed sensing^28,34^, relying on the assumption of sparsity of the measurement data.

More recently, an alternative theoretical framework for quantum state tomography based on machine learning has been put forward^35–37^, and implemented in a cold-atom experiment^38^. This approach leverages the effectiveness of unsupervised machine learning in extracting high-dimensional probability distributions from raw data^39^, combined with the high expressivity of neural networks for capturing highly-entangled quantum many-body states^40–43^. In contrast, approximate algorithms for QPT applicable to near-term quantum devices are currently lacking. While progress has been made in the context of learning non-Markovian dynamics^44,45^, the question of a scalable method capable of reconstructing noisy quantum circuits remains wide open.

In this work, we present a technique to perform QPT of quantum circuits of sizes well beyond state-of-the-art. By exploiting the structure of the problem, our approach alleviates important scaling issues of standard QPT. We combine elements of two state-of-the-art classes of algorithms, namely a tensor-network representation of a quantum channel and a data-driven global optimization inspired by unsupervised learning algorithms. The latter is in stark contrast with previous approaches where the optimisation is driven from local reconstructions on system sub-blocks^30–33^; and is key for the scalability of our method. We show numerical experiments on synthetic data for the computationally challenging case of random unitary circuits, reaching reconstruction fidelities above 0.99 for 2D 10-qubit depth-4 instances using less than 10^5^ single-shot measurements out of a tomographycally complete set of ~10^12^ settings. We also demonstrate the reconstruction of a single 5-qubit parity-check measurement in the surface code undergoing amplitude damping noise. Our proposed method paves the way to the robust and scalable verification of quantum circuits implemented in current experimental hardware.

## Results

### Quantum process tomography

**Fig. 1:**
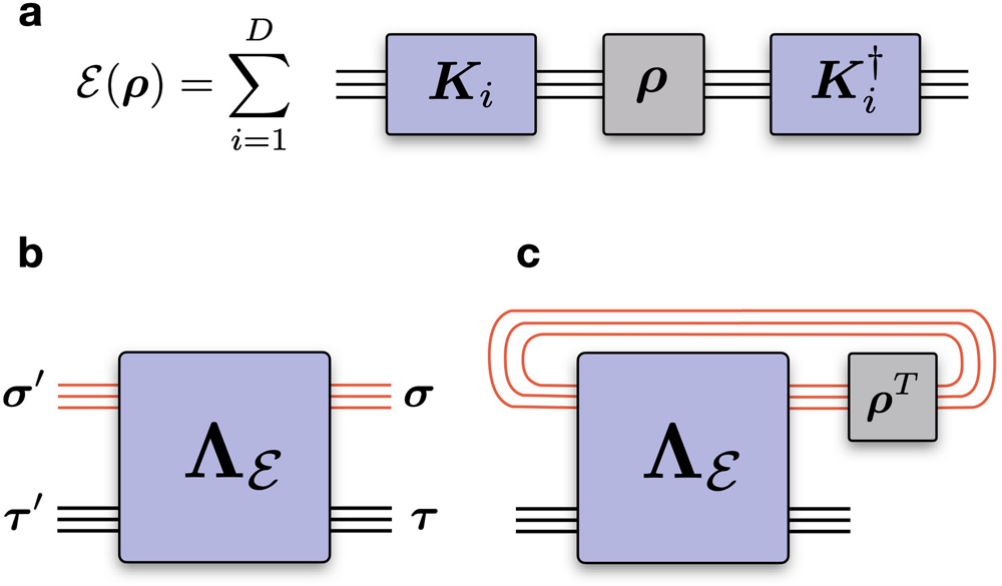
Representations of a quantum channel (with *N* = 3 qubits). **a** Evolution of a density operator ***ρ*** under a quantum channel ${{{{{{{\mathcal{E}}}}}}}}$ in the Kraus representation, where the channel has decomposition over *D* Kraus operators. **b** Representation of the channel with the Choi matrix ${{{{{{{{\boldsymbol{\Lambda }}}}}}}}}_{{{{{{{{\mathcal{E}}}}}}}}}$, a rank-4*N* tensor with the upper and lower 2*N* indices corresponding, respectively, to the input $\{\left|{{{{{{{\boldsymbol{\sigma }}}}}}}}\right\rangle \}$ and output $\{\left|{{{{{{{\boldsymbol{\tau }}}}}}}}\right\rangle \}$ Hilbert spaces. **c** Evolution of ***ρ*** using Choi representation. The output state of the channel ${{{{{{{\mathcal{E}}}}}}}}({{{{{{{\boldsymbol{\rho }}}}}}}})$ is obtained by first contracting the input space $\{\left|{{{{{{{\boldsymbol{\sigma }}}}}}}}\right\rangle \}$ with the transpose state ***ρ***^*T*^, followed by a trace, resulting into ${{{{{{{\mathcal{E}}}}}}}}({{{{{{{\boldsymbol{\rho }}}}}}}})$ over the output space $\{\left|{{{{{{{\boldsymbol{\tau }}}}}}}}\right\rangle \}$.

The unavoidable interaction of a quantum device with its environment typically introduces non-unitary dynamics in the underlying quantum state. The time evolution of the corresponding density operator ***ρ*** is generated by a *quantum channel*, a linear map ${{{{{{{\mathcal{E}}}}}}}}:\ {{{{{{{\boldsymbol{\rho }}}}}}}}\longrightarrow {{{{{{{\mathcal{E}}}}}}}}({{{{{{{\boldsymbol{\rho }}}}}}}})$ that is *completely-positive* (CP) (${{{{{{{\mathcal{E}}}}}}}}\otimes {\Bbb{1}}\ge 0$) and *trace-preserving* (TP) (${{{{{{{\rm{tr}}}}}}}}({{{{{{{\mathcal{E}}}}}}}}({{{{{{{\boldsymbol{\rho }}}}}}}}))={{{{{{{\rm{tr}}}}}}}}({{{{{{{\boldsymbol{\rho }}}}}}}})$)^46^. There are several equivalent mathematical representations of a CPTP map (see ref. ^47^ for summary). One example is the *Kraus* representation, where the channel is expressed as a set of Kraus operators {***K***_*i*_}, leading to the dynamics ${{{{{{{\mathcal{E}}}}}}}}({{{{{{{\boldsymbol{\rho }}}}}}}})={\sum }_{i}{{{{{{{{\boldsymbol{K}}}}}}}}}_{i}{{{{{{{\boldsymbol{\rho }}}}}}}}{{{{{{{{\boldsymbol{K}}}}}}}}}_{i}^{{{{\dagger}}} }$ (Fig. 1a). From the CPTP nature of the channel, it follows ${\sum }_{i}{{{{{{{{\boldsymbol{K}}}}}}}}}_{i}^{{{{\dagger}}} }{{{{{{{{\boldsymbol{K}}}}}}}}}_{i}={\Bbb{1}}$.

In the context of quantum tomography, it is natural to instead consider the *Choi matrix* of the channel^48,49^, a positive semidefinite operator

1 $${{{{{\mathbf{\Lambda}}}}}}_{{{{{{\mathcal{E}}}}}}}=\left({\mathbb{1}}\otimes{{{{{\mathcal{E}}}}}}\right) \left[\mathop{\bigotimes}\limits_{j=1}^N\left | {{\Phi}}_j^+\right\rangle\left\langle{{\Phi}}_j^+\right | \right],$$

where ${{{{{{{\mathcal{E}}}}}}}}$ is applied to one half of the tensor product of *N* unnormalized Bell pairs $|{{{\Phi }}}_{j}^{+}\rangle={\sum }_{{\sigma }_{j}={\tau }_{j}}|{\sigma }_{j}{\tau }_{j}\rangle$, and $|{\sigma }_{j}\rangle$ and $|{\tau }_{j}\rangle$ are the input and the output degrees of freedom to the channel (Fig. 1b). The channel ${{{{{{{\mathcal{E}}}}}}}}$ is CP if and only if the Choi matrix is positive-semidefinite (${{{{{{{{\boldsymbol{\Lambda }}}}}}}}}_{{{{{{{{\mathcal{E}}}}}}}}}\ge 0$). It follows that ${{{{{{{{\boldsymbol{\Lambda }}}}}}}}}_{{{{{{{{\mathcal{E}}}}}}}}}$ is isomorphic to an unnormalized density operator over an extended (bipartite) 2*N*-qubit Hilbert space (${{{{{{{\rm{Tr}}}}}}}}\,{{{{{{{{\boldsymbol{\Lambda }}}}}}}}}_{{{{{{{{\mathcal{E}}}}}}}}}={d}^{N}$, with *d* the dimension of the local Hilbert space, i.e., *d* = 2 for qubits). The TP condition of the channel ${{{{{{{\mathcal{E}}}}}}}}$ requires that the partial trace of the Choi matrix over the output indices should yield the identity over the input indices: ${{{{{{{{\rm{Tr}}}}}}}}}_{{{{{{{{\boldsymbol{\tau }}}}}}}}}\,{{{{{{{{\boldsymbol{\Lambda }}}}}}}}}_{{{{{{{{\mathcal{E}}}}}}}}}={{\mathbb{1}}}_{{{{{{{{\boldsymbol{\sigma }}}}}}}}}$^47^. The evolution of a generic quantum state ***ρ*** under the channel ${{{{{{{\mathcal{E}}}}}}}}$ is obtained through the Choi matrix as^47^ (Fig. 1c)

2 $${{{{{{{\mathcal{E}}}}}}}}({{{{{{{\boldsymbol{\rho }}}}}}}})={{{{{{{{\rm{Tr}}}}}}}}}_{{{{{{{{\boldsymbol{\sigma }}}}}}}}}\,\left[({{{{{{{{\boldsymbol{\rho }}}}}}}}}^{T}\otimes {{\Bbb{1}}}_{{{{{{{{\boldsymbol{\tau }}}}}}}}})\,{{{{{{{{\boldsymbol{\Lambda }}}}}}}}}_{{{{{{{{\mathcal{E}}}}}}}}}\right],$$

where ***ρ***^*T*^ denotes matrix transposition.

Unlike the Kraus representation, the Choi matrix of a quantum channel is unique. This implies that QPT simply accounts of fitting the matrix elements of ${{{{{{{{\boldsymbol{\Lambda }}}}}}}}}_{{{{{{{{\mathcal{E}}}}}}}}}$ to the data, which consists of a special set of prepared input states to the channel and a set of measurements on the output states. In particular, a set of input states and measurements is called *informationally-complete* (IC) if the inputs {***ρ***_***α***_} and the measurement operators {***M***_***β***_} span in full the input and the output Hilbert spaces of the quantum channel, respectively. In this case, the probability distribution

3 $${P}_{{{{{{{{\mathcal{E}}}}}}}}}({{{{{{{\boldsymbol{\beta }}}}}}}}|{{{{{{{\boldsymbol{\alpha }}}}}}}}) 	={{{{{{{{\rm{Tr}}}}}}}}}_{{{{{{{{\boldsymbol{\tau }}}}}}}}}\left[{{{{{{{{\boldsymbol{M}}}}}}}}}_{{{{{{{{\boldsymbol{\beta }}}}}}}}}{{{{{{{\mathcal{E}}}}}}}}({{{{{{{\boldsymbol{{\rho }}}}}}}_{{{{{{{{\boldsymbol{\alpha }}}}}}}}}}})\right]\\ 	={{{{{{{{\rm{Tr}}}}}}}}}_{{{{{{{{\boldsymbol{\tau }}}}}}}},{{{{{{{\boldsymbol{\sigma }}}}}}}}}\left[({{{{{{{{\boldsymbol{\rho }}}}}}}}}_{{{{{{{{\boldsymbol{\alpha }}}}}}}}}^{T}\otimes {{{{{{{{\boldsymbol{M}}}}}}}}}_{{{{{{{{\boldsymbol{\beta }}}}}}}}})\,{{{{{{{{\boldsymbol{\Lambda }}}}}}}}}_{{{{{{{{\mathcal{E}}}}}}}}}\right]$$

that a measurement on the output state ${{{{{{{\mathcal{E}}}}}}}}({{{{{{{{\boldsymbol{\rho }}}}}}}}}_{{{{{{{{\boldsymbol{\alpha }}}}}}}}})$ of the channel applied to the input state ***ρ***_***α***_ yields outcome ***M***_***β***_ contains complete information on the channel. That is, ${P}_{{{{{{{{\mathcal{E}}}}}}}}}\,({{{{{{{\boldsymbol{\beta }}}}}}}}|{{{{{{{\boldsymbol{\alpha }}}}}}}})$ uniquely characterizes the channel, and can be used to reconstruct the corresponding (unknown) Choi matrix ${{{{{{{{\boldsymbol{\Lambda }}}}}}}}}_{{{{{{{{\mathcal{E}}}}}}}}}$.

The standard approach to perform QPT consists of parametrizing the Choi matrix (i.e., using a 4^*N*^ × 4^*N*^ matrix) and extracting its elements by solving the maximum likelihood estimation problem^22^. There are two fundamental limitations of this approach. First, it requires the parametrization of the full Choi matrix, which scales exponentially with the number of qubits. Second, in order to achieve a high-fidelity fit, the full IC set of input states and measurements is required, which also scales exponentially with *N*. For these reasons, full QPT has remained restricted to very small system sizes.

### Tensor-network Choi matrix

**Fig. 2:**
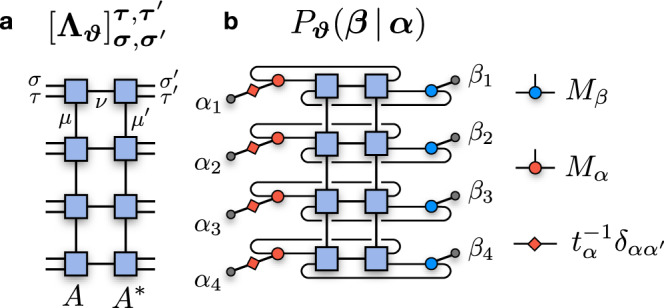
Quantum process tomography with tensor networks. **a** The quantum process (*N* = 4) is represented by a Choi matrix **Λ**_***ϑ***_, parametrized by a locally-purified density operator (LPDO). The input and output indices of the process are {*σ*_*j*_} and {*τ*_*j*_}, respectively. **b** Tensor contraction evaluating the conditional probability distribution *P*_***ϑ***_(***β***∣***α***), i.e., the probability that the LPDO Choi matrix associates with the measurement ***M***_***β***_ given the state ${{{{{{{{\boldsymbol{\rho }}}}}}}}}_{{{{{{{{\boldsymbol{\alpha }}}}}}}}}={t}_{{{{{{{{\boldsymbol{\alpha }}}}}}}}}^{-1}{{{{{{{{\boldsymbol{M}}}}}}}}}_{{{{{{{{\boldsymbol{\alpha }}}}}}}}}$ at the input of the channel.

In order to mitigate the exponential complexity of full QPT, we first introduce an efficient representation of Choi matrices in terms of tensor networks, whose total number of parameters is small compared to the dimension of the process Hilbert space. Specifically, we consider a parametrization of the Choi matrix **Λ**_***ϑ***_ (with ***ϑ*** the set of variational parameters) in terms of locally-purified density operator (LPDO), a class of matrix product operators that are non-negative by construction^50^ (Fig. 2a). Given a basis for the input $\{\left|{{{{{{{\boldsymbol{\sigma }}}}}}}}\right\rangle \}$ and the output $\{\left|{{{{{{{\boldsymbol{\tau }}}}}}}}\right\rangle \}$ Hilbert spaces, the matrix elements $\langle {{{{{{{\boldsymbol{\sigma }}}}}}}},{{{{{{{\boldsymbol{\tau }}}}}}}}|{{{{{{{{\boldsymbol{\Lambda }}}}}}}}}_{{{{{{{{\boldsymbol{\vartheta }}}}}}}}}|{{{{{{{{\boldsymbol{\sigma }}}}}}}}}^{{\prime} },{{{{{{{{\boldsymbol{\tau }}}}}}}}}^{{\prime} }\rangle$ of the LPDO Choi matrix are given by

4 $${[{{{{{{{{\boldsymbol{\Lambda }}}}}}}}}_{{{{{{{{\boldsymbol{\vartheta }}}}}}}}}]}_{{{{{{{{\boldsymbol{\sigma }}}}}}}},{{{{{{{{\boldsymbol{\sigma }}}}}}}}}^{{\prime} }}^{{{{{{{{\boldsymbol{\tau }}}}}}}},{{{{{{{{\boldsymbol{\tau }}}}}}}}}^{{\prime} }}=\mathop{\sum}\limits_{\{{{{{{{{\boldsymbol{\mu }}}}}}}},{{{{{{{{\boldsymbol{\mu }}}}}}}}}^{{\prime} }\}}\mathop{\sum}\limits_{\{{{{{{{{\boldsymbol{\nu }}}}}}}}\}}\mathop{\prod }\limits_{j=1}^{N}{[{A}_{j}]}_{{\mu }_{j-1},{\nu }_{j},{\mu }_{j}}^{{\tau }_{j},{\sigma }_{j}}{[{A}_{j}^{*}]}_{{\mu }_{j-1}^{{\prime} },{\nu }_{j},{\mu }_{j}^{{\prime} }}^{{\tau }_{j}^{{\prime} },{\sigma }_{j}^{{\prime} }},$$

where ***ϑ*** = {*A*_*j*_}. Here, we assume that {*A*_*j*_} already incorporate the proper normalization ${{{{{{{{\rm{Tr}}}}}}}}}_{{{{{{{{\boldsymbol{\sigma }}}}}}}},{{{{{{{\boldsymbol{\tau }}}}}}}}}{{{{{{{{\boldsymbol{\Lambda }}}}}}}}}_{{{{{{{{\boldsymbol{\vartheta }}}}}}}}}={d}^{N}$. Each tensor *A*_*j*_ has input index *σ*_*j*_, output index *τ*_*j*_, *bond indices* (*μ*_*j*−1_, *μ*_*j*_) and *Kraus indexν*_*j*_. The bond and Kraus dimensions of the LPDO are defined as ${\chi }_{\mu }={\max }_{j}\{{\chi }_{{\mu }_{j}}=\dim [{\mu }_{j}]\}$ and ${\chi }_{\nu }={\max }_{j}\{{\chi }_{{\nu }_{j}}=\dim [{\nu }_{j}]\}$. By setting *χ*_*ν*_ = 1, the resulting Choi matrix is rank-1, ${{{{{{{{\boldsymbol{\Lambda }}}}}}}}}_{{{{{{{{\boldsymbol{\vartheta }}}}}}}}}=\left|{{{{{{{{\boldsymbol{\Psi }}}}}}}}}_{{{{{{{{\boldsymbol{\vartheta }}}}}}}}}\right\rangle \,\left\langle {{{{{{{{\boldsymbol{\Psi }}}}}}}}}_{{{{{{{{\boldsymbol{\vartheta }}}}}}}}}\right|$, where $\left|{{{{{{{{\boldsymbol{\Psi }}}}}}}}}_{{{{{{{{\boldsymbol{\vartheta }}}}}}}}}\right\rangle$ is an MPS with physical dimension *d*^2^ and bond dimension *χ*_*μ*_. This corresponds to ${{{{{{{\mathcal{E}}}}}}}}$ being a unitary channel.

### QPT via unsupervised learning

To perform process tomography with LPDOs, we consider the standard QPT setup of positive operator valued measures (POVM) ${{{{{{{{\boldsymbol{M}}}}}}}}}_{{{{{{{{\boldsymbol{\beta }}}}}}}}}{=\bigotimes }_{j=1}^{N}{M}_{{\beta }_{j}}$, where ${\{{M}_{{\beta }_{j}}\}}_{{\beta }_{j}=1}^{{K}_{m}}$ are single-qubit POVMs with *K*_*m*_ measurement outcomes (${M}_{{\beta }_{j}}\ge 0$ and ${\sum }_{{\beta }_{j}}{M}_{{\beta }_{j}}={{\Bbb{1}}}_{j}$). As input states to the channel, we take product states ${{{{{{{{\boldsymbol{\rho }}}}}}}}}_{{{{{{{{\boldsymbol{\alpha }}}}}}}}}{=\bigotimes }_{j=1}^{N}{\rho }_{{\alpha }_{j}}$. The preparation states and output measurements are identified by the classical strings ***α*** = (*α*_1_, …, *α*_*N*_) and ***β*** = (*β*_1_, …, *β*_*N*_), respectively. In the following, we use for convenience the same POVM set for the input states ${{{{{{{{\boldsymbol{\rho }}}}}}}}}_{{{{{{{{\boldsymbol{\alpha }}}}}}}}}={t}_{{{{{{{{\boldsymbol{\alpha }}}}}}}}}^{-1}{{{{{{{{\boldsymbol{M}}}}}}}}}_{{{{{{{{\boldsymbol{\alpha }}}}}}}}}$ (i.e., *K*_*m*_ = *K*_*p*_ ≡ *K*), where ${t}_{{{{{{{{\boldsymbol{\alpha }}}}}}}}}={{{{{{{\rm{Tr}}}}}}}}\,{{{{{{{{\boldsymbol{M}}}}}}}}}_{{{{{{{{\boldsymbol{\alpha }}}}}}}}}={\prod }_{j}{{{{{{{\rm{Tr}}}}}}}}\,{M}_{{\alpha }_{j}}$ is a normalization factor.

We generate a training data set by first preparing a finite set of *M* input states ${\{{{{{{{{{\boldsymbol{\rho }}}}}}}}}_{{{{{{{{\boldsymbol{\alpha }}}}}}}}}^{(k)}\}}_{k=1}^{M}$, randomly sampled according to a fixed prior distribution *Q*(***α***). We then apply the channel to each state, and perform a measurement at its output, recording the outcomes ${\{{{{{{{{{\boldsymbol{M}}}}}}}}}_{{{{{{{{\boldsymbol{\beta }}}}}}}}}^{(k)}\}}_{\!k=1}^{\!M}$. The resulting data set is specified by *M* strings of 2*NK*-valued integers, ${{{{{{{\mathcal{D}}}}}}}}={\{({{{{{{{{\boldsymbol{\alpha }}}}}}}}}^{(k)},{{{{{{{{\boldsymbol{\beta }}}}}}}}}^{(k)})\}}_{\!k=1}^{\!M}$, with joint probability distribution ${P}_{{{{{{{{\mathcal{D}}}}}}}}}({{{{{{{\boldsymbol{\alpha }}}}}}}},{{{{{{{\boldsymbol{\beta }}}}}}}})=Q({{{{{{{\boldsymbol{\alpha }}}}}}}}){P}_{{{{{{{{\mathcal{E}}}}}}}}}({{{{{{{\boldsymbol{\beta }}}}}}}}\,|\,{{{{{{{\boldsymbol{\alpha }}}}}}}})$. Similarly, we can estimate the corresponding probability distribution *P*_***ϑ***_(***β***∣***α***) for the Choi matrix **Λ**_***ϑ***_. Since both input states and output POVMs factorize over the extended Hilbert space, estimating the probability translates into local contractions of the tensors *A*_*j*_ with the tensor product ${\rho }_{{\alpha }_{j}}^{T}\otimes {M}_{{\beta }_{j}}$ at all sites *j* (Fig. 2b). The cost of this operation is ${{{{{{{\mathcal{O}}}}}}}}({d}^{2}N{\chi }_{\nu }{\chi }_{\mu }^{3})$, remaining efficient as long as the bond dimensions (*χ*_*μ*_, *χ*_*ν*_) are sufficiently small.

The learning procedure, inspired by generative modeling of neural networks in machine learning applications^39^, consists of varying the parameters ***ϑ*** to minimize the distance between the LPDO distribution *P*_***ϑ***_(***β***∣***α***) and the target distribution ${P}_{{{{{{{{\mathcal{E}}}}}}}}}({{{{{{{\boldsymbol{\beta }}}}}}}}\,|\,{{{{{{{\boldsymbol{\alpha }}}}}}}})$, averaged over the input prior *Q*(***α***). As distance, we adopt the Kullbach-Leibler divergence^51^:

5 $${D}_{KL}=\mathop{\sum}\limits_{\{{{{{{{{\boldsymbol{\alpha }}}}}}}}\}}Q({{{{{{{\boldsymbol{\alpha }}}}}}}})\mathop{\sum}\limits_{\{{{{{{{{\boldsymbol{\beta }}}}}}}}\}}{P}_{{{{{{{{\mathcal{E}}}}}}}}}({{{{{{{\boldsymbol{\beta }}}}}}}}\,|\,{{{{{{{\boldsymbol{\alpha }}}}}}}})\log \frac{{P}_{{{{{{{{\mathcal{E}}}}}}}}}({{{{{{{\boldsymbol{\beta }}}}}}}}\,|\,{{{{{{{\boldsymbol{\alpha }}}}}}}})}{{P}_{{{{{{{{\boldsymbol{\vartheta }}}}}}}}}({{{{{{{\boldsymbol{\beta }}}}}}}}\,|\,{{{{{{{\boldsymbol{\alpha }}}}}}}})},$$

Minimizing this quantity is equivalent to minimizing the negative log-likelihood

6 $${{{{{{{\mathcal{C}}}}}}}}({{{{{{{\boldsymbol{\vartheta }}}}}}}})=-\frac{1}{M}\mathop{\sum }\limits_{k=1}^{M}\log {P}_{{{{{{{{\boldsymbol{\vartheta }}}}}}}}}({{{{{{{{\boldsymbol{\beta }}}}}}}}}_{k}\,|\,{{{{{{{{\boldsymbol{\alpha }}}}}}}}}_{k}),$$

where the average is taken over the data set ${{{{{{{\mathcal{D}}}}}}}}$. This is the cost function of our optimization problem. This type of tensor network optimization, also explored for quantum state tomography^52^, is in contrast with the local optimization used in the original formulation of MPS tomography, which relies on measurements of local subsystems and entails and exponential scaling with the size of the subsystems^30,33^.

The LPDO parameters are iteratively updated using gradient descent ${{{{{{{\boldsymbol{\vartheta }}}}}}}}\to {{{{{{{\boldsymbol{\vartheta }}}}}}}}-\eta {\nabla }_{{{{{{{{\boldsymbol{\vartheta }}}}}}}}}{{{{{{{\mathcal{C}}}}}}}}({{{{{{{\boldsymbol{\vartheta }}}}}}}})$ (or a variation thereof), where *η* is the size of the gradient update (i.e., the *learning rate*). In our simulations, we optimize the LPDO using automatic differentiation software^53^, a framework that is being increasingly explored in tensor networks applications^54,55^. However, the gradients of the cost function can also be derived analytically^56,57^, and are shown in the Supplementary Material. We also point out that, due to the tensor-network parametrization of the Choi matrix, the optimization landscape is non-convex, which means that there is no a priori guarantee that the training will yield the exact target Choi matrix in the limit of infinite data.

In defining our parametrized model **Λ**_***ϑ***_, we exploited the fact that Choi matrices are isomorphic to density operators, which justifies the use of LPDOs. However, while ${{{{{{{{\boldsymbol{\Lambda }}}}}}}}}_{{{{{{{{\boldsymbol{\vartheta }}}}}}}}}={{{{{{{{\boldsymbol{\Lambda }}}}}}}}}_{{{{{{{{\boldsymbol{\vartheta }}}}}}}}}^{{{{\dagger}}} }$ and **Λ**_***ϑ***_ ≥ 0 by construction, the LPDO is not inherently TP. That is, the condition ${{{{{{{{\rm{Tr}}}}}}}}}_{{{{{{{{\boldsymbol{\tau }}}}}}}}}\,{{{{{{{{\boldsymbol{\Lambda }}}}}}}}}_{{{{{{{{\boldsymbol{\vartheta }}}}}}}}}={{\Bbb{1}}}_{{{{{{{{\boldsymbol{\sigma }}}}}}}}}$ is not enforced at the level of the elementary tensors {*A*_*j*_}. We expect that, if *M* is large enough and the model faithfully learns the quantum channel underlying the training data set, this property should also be approximately satisfied. Nonetheless, we can approximately impose the TP constraint by adding a *regularization* term to ${{{{{{{\mathcal{C}}}}}}}}({{{{{{{\boldsymbol{\vartheta }}}}}}}})$, which induces a bias towards trace-preserving matrices. We define this regularization term as

7 $${{{\Gamma }}}_{{{{{{{{\boldsymbol{\vartheta }}}}}}}}}=\sqrt{{d}^{-N}}\parallel {{{{{{{{\mathbf{\Delta }}}}}}}}}_{{{{{{{{\boldsymbol{\vartheta }}}}}}}}}{\parallel }_{F}=\sqrt{{d}^{-N}}\sqrt{{{{{{{{{\rm{Tr}}}}}}}}}_{{{{{{{{\boldsymbol{\sigma }}}}}}}}}\left({{{{{{{{\mathbf{\Delta }}}}}}}}}_{{{{{{{{\boldsymbol{\vartheta }}}}}}}}}{{{{{{{{\mathbf{\Delta }}}}}}}}}_{{{{{{{{\boldsymbol{\vartheta }}}}}}}}}^{{{{\dagger}}} }\right)},$$

where ${{{{{{{{\mathbf{\Delta }}}}}}}}}_{{{{{{{{\boldsymbol{\vartheta }}}}}}}}}={{{{{{{{\rm{Tr}}}}}}}}}_{{{{{{{{\boldsymbol{\tau }}}}}}}}}{{{{{{{{\boldsymbol{\Lambda }}}}}}}}}_{{{{{{{{\boldsymbol{\vartheta }}}}}}}}}-{{\Bbb{1}}}_{{{{{{{{\boldsymbol{\sigma }}}}}}}}}$. The final cost function becomes ${{{{{{{\mathcal{C}}}}}}}}({{{{{{{\boldsymbol{\vartheta }}}}}}}})=-{\langle \log {P}_{{{{{{{{\boldsymbol{\vartheta }}}}}}}}}({{{{{{{\boldsymbol{\beta }}}}}}}}|{{{{{{{\boldsymbol{\alpha }}}}}}}})\rangle }_{{{{{{{{\mathcal{D}}}}}}}}}+\kappa \,{{{\Gamma }}}_{{{{{{{{\boldsymbol{\vartheta }}}}}}}}}$, where *κ* is a hyper-parameter of the optimization.

### Numerical experiments

We study the performance of LPDO-based QPT for unitary and noisy quantum channels. We adopt, for both the input states and measurements, the POVM set built out of the rank-1 projectors of the *K* = 6 eigenstates of the Pauli matrices. This POVM is informationally over-complete and experimentally-friendly, as it can be implemented with routinely available single-qubit measurements. For all the instances described, we generate the training data set ${{{{{{{\mathcal{D}}}}}}}}$ using a uniform prior distribution *Q*(***α***) = *K*^−*N*^. We split the data set into a training set and a validation set, containing, respectively, 80% and 20% of the total data. The training data set contains the measurements used to compute the gradients and train the LPDO. The remaining held-out data is used for cross-validation for selecting the optimal model. That is, the cost function computed on the validation data set is used to verify that the model is not overfitting the training data set, and to choose the optimal training epoch (see “Methods”). Details on the data generation and the LPDO trainings are provided in the Supplementary Material.

We start by studying the case of a unitary channel characterized by a rank-1 Choi matrix ${{{{{{{{\boldsymbol{\Lambda }}}}}}}}}_{{{{{{{{\mathcal{E}}}}}}}}}=\left|{{{{{{{{\boldsymbol{\Psi }}}}}}}}}_{{{{{{{{\mathcal{E}}}}}}}}}\right\rangle \,\left\langle {{{{{{{{\boldsymbol{\Psi }}}}}}}}}_{{{{{{{{\mathcal{E}}}}}}}}}\right|$. We perform QPT by setting the Kraus dimension to *χ*_*ν*_ = 1, leading to the parametrized Choi matrix ${{{{{{{{\boldsymbol{\Lambda }}}}}}}}}_{{{{{{{{\boldsymbol{\vartheta }}}}}}}}}=\left|{{{{{{{{\boldsymbol{\Psi }}}}}}}}}_{{{{{{{{\boldsymbol{\vartheta }}}}}}}}}\right\rangle \,\left\langle {{{{{{{{\boldsymbol{\Psi }}}}}}}}}_{{{{{{{{\boldsymbol{\vartheta }}}}}}}}}\right|$ expressed in terms of an MPS **Ψ**_***ϑ***_. We also set the bond dimension of the LPDO *χ*_*μ*_ equal to the bond dimension ${\chi }_{{{{{{{{\mathcal{E}}}}}}}}}$ of ${{{{{{{{\boldsymbol{\Psi }}}}}}}}}_{{{{{{{{\mathcal{E}}}}}}}}}$. Thus, there is no approximation in the representation of the channel, and any reconstruction error generates solely from the finite size of the data set and any potential inefficiency of the optimization procedure. We point out that, when the ideal target quantum circuit is known, it is possible to estimate what is the minimum value of ${\chi }_{{{{{{{{\mathcal{E}}}}}}}}}$ leading to a faithful tensor-network representation of the quantum circuit. Both conditions on *χ*_*μ*_ and *χ*_*ν*_ will be lifted for the reconstruction of a noisy channel, later in this section.

During the training, we measure the cost function computed on both the training and validation data sets. The former monitors the learning progress, while the latter monitors the overfitting and is used to select the optimal parameters (as those in the training epoch that minimize the validation cost function). In addition, we also measure the *quantum process fidelity* ${{{{{{{\mathcal{F}}}}}}}}({{{{{{{{\boldsymbol{\Lambda }}}}}}}}}_{{{{{{{{\mathcal{E}}}}}}}}},{{{{{{{{\boldsymbol{\Lambda }}}}}}}}}_{{{{{{{{\boldsymbol{\vartheta }}}}}}}}})$ of the reconstruction to the true channel used to generate the data, which measures the average-case performance of the reconstruction. The process fidelity is equivalent to the quantum state fidelity between the two (properly normalized) Choi matrices

8 $${{{{{{{\mathcal{F}}}}}}}}({{{{{{{{\boldsymbol{\Lambda }}}}}}}}}_{{{{{{{{\mathcal{E}}}}}}}}},{{{{{{{{\boldsymbol{\Lambda }}}}}}}}}_{{{{{{{{\boldsymbol{\vartheta }}}}}}}}})={d}^{-2N}{\left({{{{{{{\rm{Tr}}}}}}}}\sqrt{\sqrt{{{{{{{{{\boldsymbol{\Lambda }}}}}}}}}_{{{{{{{{\mathcal{E}}}}}}}}}}{{{{{{{{\boldsymbol{\Lambda }}}}}}}}}_{{{{{{{{\boldsymbol{\vartheta }}}}}}}}}\sqrt{{{{{{{{{\boldsymbol{\Lambda }}}}}}}}}_{{{{{{{{\mathcal{E}}}}}}}}}}}\right)}^{2},$$

which reduces to ${{{{{{{\mathcal{F}}}}}}}}({{{{{{{{\boldsymbol{\Lambda }}}}}}}}}_{{{{{{{{\boldsymbol{\vartheta }}}}}}}}},{{{{{{{{\boldsymbol{\Lambda }}}}}}}}}_{{{{{{{{\mathcal{E}}}}}}}}})={d}^{-2N}\langle {{{{{{{{\boldsymbol{\Psi }}}}}}}}}_{{{{{{{{\mathcal{E}}}}}}}}}|{{{{{{{{\boldsymbol{\Lambda }}}}}}}}}_{{{{{{{{\boldsymbol{\vartheta }}}}}}}}}|{{{{{{{{\boldsymbol{\Psi }}}}}}}}}_{{{{{{{{\mathcal{E}}}}}}}}}\rangle$ when the target Choi matrix ${{{{{{{{\boldsymbol{\Lambda }}}}}}}}}_{{{{{{{{\mathcal{E}}}}}}}}}$ is rank-1. In addition, in the latter rank-1 case, the process fidelity also directly gives other process-closeness quantifiers, such as the Frobenius-norm distance between the Choi states in question^58^. We note that—being an average-case metric—the process fidelity can lead to reconstruction error estimates that may differ from worst-case estimates (as quantified, for instance, by the diamond-norm distance) by orders of magnitude (see e.g., ref. ^59^ and refs therein). Nevertheless, the fidelity is significantly easier to estimate than other more stringent metrics, which makes it one of the most practical and commonly used metric for experimental state or process reconstruction.

In the numerical experiments, we show the fit fidelity between the target and the learned Choi states, since it provides a direct measure of the quality of the tomographic reconstruction. However, QPT is typically used to extract valuable information about a device, rather than returning a single figure of merit (which can be obtained with more efficient methods^16^). One important example is whether the Choi state factorizes over a specific partition of the device. Within our framework, this can be easily checked in a scalable manner by simply tracing out local tensors in the Choi LPDO (if averaging over both input and output states).

**Fig. 3:**
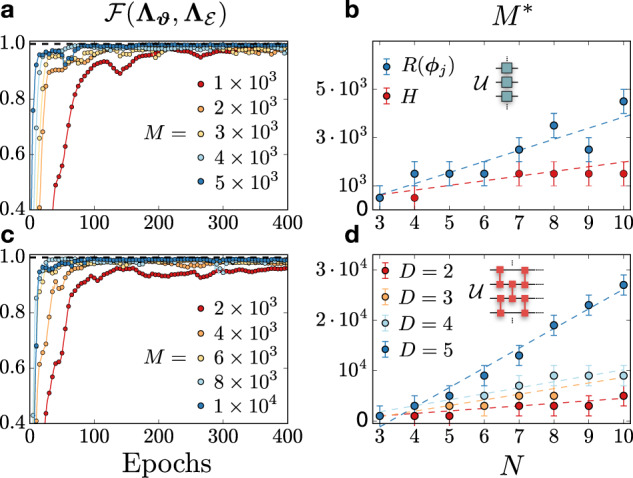
Process reconstruction for unitary quantum circuits containing single-qubit and two-qubit quantum gates. **a** Reconstruction fidelity during the LPDO training for a circuits with *N* = 4 qubits containing a single layer of Hadamard gates. Different curves corresponds to an increasing size *M* of the data set. **b** Scaling of the minimum number of samples M* as a function of *N* to reach a reconstruction infidelity of *ε* = 0.025 (i.e., the sample complexity) for a circuit with Hadamard gates (red) and a circuit with random single-qubit rotations *R*(***φ***_*j*_) (blue). **c** Reconstruction fidelity for a circuit with *N* = 4 qubits containing 4 layers of controlled-not (CX) gates, for various data set sizes *M*. **d** Sample complexity for quantum circuits with different depths *D* containing layers of CX gates. For the sample complexity plots, the value M* is obtained by sequentially increasing *M* until the threshold in accuracy is met. Error bars are given by the step-size in *M*, and dashed lines are linear fits.

The first test-case is a unitary quantum circuit containing a single layer of Hadamard gates acting on all qubits. We train LPDOs for different sizes *M* of the training data set, and we show in Fig. 3a the corresponding reconstruction fidelities measured at each training iteration (*epoch*), for *N* = 4 qubits. From this data, we can compute the minimum number of training samples M* required to reach a fixed accuracy *ε* in the reconstruction infidelity $1-{{{{{{{\mathcal{F}}}}}}}}({{{{{{{{\boldsymbol{\Lambda }}}}}}}}}_{{{{{{{{\boldsymbol{\vartheta }}}}}}}}},{{{{{{{{\boldsymbol{\Lambda }}}}}}}}}_{{{{{{{{\mathcal{E}}}}}}}}})$. By repeating the same experiment for several systems sizes up to *N* = 10 (with *ε* = 0.025), we show the sample complexity in Fig. 3b—the value M* as a function of *N*—observing a favorable scaling consistent with a linear behavior. We repeat the same experiment for a single layer of random single-qubit rotations *R*(***φ***_*j*_), observing a similar scaling with a steeper slope.

We also consider a quantum circuit containing *D* layers of controlled-NOT (CX) gates applied between neighboring qubits in a one-dimensional geometry. Each layer is applied in a staggered manner (inset of Fig. 3d). We perform the same analysis as for the one-qubit circuits, and plot the fidelity curves for various *M* for a circuit with *N* = 4 qubits and depth *D* = 4 (Fig. 3c). The sample complexity, computed in an analogous manner, is shown in Fig. 3d for different depths *D*. As expected, the threshold M* increases with the depth of the circuit.

**Fig. 4:**
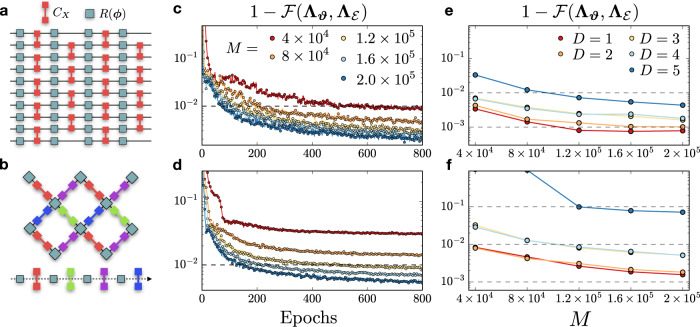
Random quantum circuits. **a** One-dimensional quantum circuit with *N* = 10 qubits and *D* = 4 layers, each one consisting of random single-qubit rotations and CX gates, the latter applied in a staggered pattern between even and odd layers. **b** Two-dimensional random quantum circuit, where each layer applies random single-qubit rotations and CX gates according to the colored sequence shown at the bottom of the image. In the panels (**c**) and (**d**), we show the reconstruction infidelity at each epoch, respectively, for a one- and two-dimensional quantum circuit with depth *D* = 4, for various data set sizes *M*. Subplots (**e**) and (**f**) show the lowest infidelities, obtained via cross-validation on held-out data, as a function of the data set size *M* for different depths.

We now move to the more challenging case of 10-qubit random quantum circuits with depth *D*, for both one- and two-dimensional qubit arrays. Each layer in the circuit consists of *N* random single-qubit rotations followed by a layer of CX gates. For the one-dimensional circuit, the CX gates alternate between even and odd layers (Fig. 4a). For the two-dimensional circuit, the CX gates are applied in a sequence according to the colors shows in Fig. 4b. In the plots of Fig. 4c, d we show the process infidelity during the training for depth-4 circuits and different values of the data set size *M*. We observe that, with enough number of single-shot samples *M*, the reconstructions surpass a fidelity of ${{{{{{{\mathcal{F}}}}}}}}=0.99$.

We evaluate the optimal LPDO parameters using cross-validation on the held-out data, a metric that does not rely on any prior information about the process and available in an experimental setting. We show in Fig. 4e, f the corresponding lowest infidelities obtained during the training as a function of *M*. As in the previous case, the number of samples to reach a given accuracy increases with the depth of the circuit. For the one-dimensional circuit, the fidelity reaches ${{{{{{{\mathcal{F}}}}}}}}\, > \,0.99$ with 4 × 10^4^ measurements up to *D* = 4, and converges to ${{{{{{{\mathcal{F}}}}}}}}\,\approx \,0.999$ and ${{{{{{{\mathcal{F}}}}}}}}\,\approx \,0.998$ for *D* = 2 and *D* = 4, respectively. For the two-dimensional circuit, the fidelity converges to ${{{{{{{\mathcal{F}}}}}}}}\, > \,0.99$ up to *D* = 4 at *M* = 2 × 10^5^, while ${{{{{{{\mathcal{F}}}}}}}}\,\approx \,0.93$ for *D* = 5. In this case, the bond dimension of the target circuit is *χ*_*μ*_ = 32, a four-fold increase from *χ*_*μ*_ = 8 of the *D* = 4 circuit. We emphasize that the size *M* of the data set used is very small in comparison with any IC set of input states and measurement settings. For instance, for our choice of POVMs, the total number of experimental configurations for a 10-qubit circuit is 6^*N*^3^*N*^ ~ 10^12^.

**Fig. 5:**
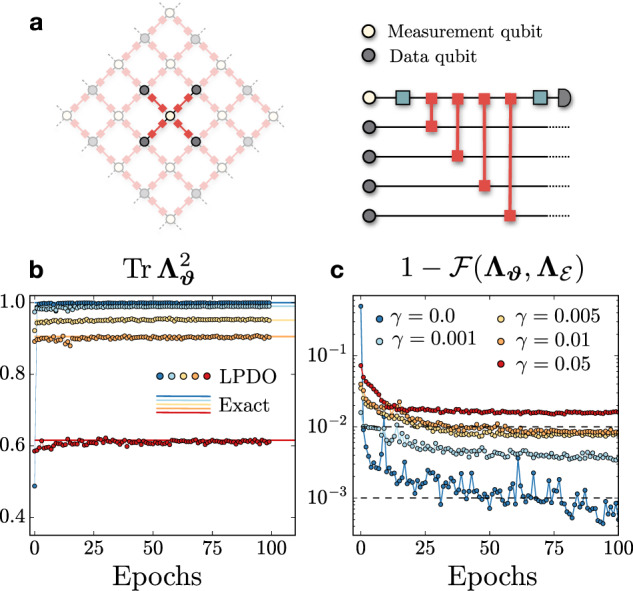
Noisy stabilizer in the surface code. **a** A *X*-stabilizer plaquette embedded into the surface code (left) and the quantum circuit performing the parity-check measurement (right), containing Hadamard and CX gates. **b** Purity of the LPDO Choi matrix during training (markers), compared to the exact Choi matrix (solid lines). **c** Infidelity measurement during training for a data set size of *M* = 5 × 10^5^ single-shot measurement outcomes.

Finally, we turn to the case of a quantum circuit undergoing a noise channel. As a test case, we study a single *X*-stabilizer measurement of the surface code, a paradigmatic model of topological quantum computation^60,61^. The circuit contains a total of *N* = 5 qubits, where a single measurement qubit is used to stabilize the *X* parity-check between four data qubits. The quantum circuit for the stabilizer measurement consists of a Hadamard gate on the measurement qubit, four CX gates between the measurement qubit and each data qubit, followed by an additional Hadamard gate on the measurement qubit (Fig. 5a). We apply a single-qubit amplitude damping channel to each qubit involved in a quantum gate after its application, with a fixed decay probability *γ* ∈ [0, …, 0.05].

We perform the reconstruction by varying both the bond dimension and the Kraus dimension, until convergence is found, and we show the results for *χ*_*μ*_ = *χ*_*ν*_ = 6. During the training, we measure the reconstruction fidelity, as well as the purity ${{{{{{{\rm{Tr}}}}}}}}\,{{{{{{{{\boldsymbol{\Lambda }}}}}}}}}_{{{{{{{{\boldsymbol{\vartheta }}}}}}}}}^{2}$ of the LPDO. For all values of the decay probability *γ*, we observe that the purity converges to the correct value (solid lines) computed from the exact Choi matrix (Fig. 5b), suggesting that the Kraus dimension of the LPDOs is sufficient to capture the target noisy channel. We also show the process infidelity curves obtained using a total of *M* = 5 × 10^5^ measurement samples, for different values of *γ* (Fig. 5c). While for the noiseless channel the fidelity reaches ${{{{{{{\mathcal{F}}}}}}}}\, > \,0.999$, the learning appears to become increasingly harder for larger values of *γ*. The lowest fidelity ${{{{{{{\mathcal{F}}}}}}}}\,\approx \,0.985$ is found at *γ* = 0.05, which is a fairly large decay probability for current experiments. For lower levels of noise, the reconstruction reaches remarkably high fidelities ${{{{{{{\mathcal{F}}}}}}}}\, > \,0.99$.

## Discussion

We introduced a procedure for quantum process tomography that integrates a tensor network representation of the Choi matrix in terms of a locally-purified matrix product operator^50^, and an optimization strategy based on by machine learning algorithms for generative modeling of high-dimensional probability distributions^39^. We demonstrated the power and scalability of the technique using simulated data for unitary random quantum circuits, reaching system sizes of up to 10 qubits and depth 5, and a stabilizer measurement of the surface code undergoing amplitude damping noise. In both cases, the resulting process fidelities reach values close to ${{{{{{{\mathcal{F}}}}}}}}=0.99$, using single-shot samples corresponding to a small fraction of the total number of preparation and measurements in the corresponding informationally-complete set, amenable to current experiments.

Due to the entanglement structure induced by a tensor network with small bond dimension, our technique lends itself extremely well to the characterization of quantum hardware operating circuits of sufficiently low depth. The stringent limitation of standard process tomography in the accessible number of qubits is lifted, allowing the reconstruction of large quantum circuits for the case of one-dimensional geometries, as well as two-dimensional thin strips.

Our work demonstrates how infusing state-of-the-art tensor network algorithms with machine learning ideas has the potential to unlock progress in the validation and characterization of currently available quantum devices, and in the design of better error mitigation protocols. This combination makes our techniques relevant for tackling several key obstacles to realizing large-scale quantum computation, including the need for quantum error correction and fault tolerance, which naturally calls for the systematic characterization of effective error terms in large quantum circuits such as the ones studied here.

We anticipate that our strategy will enable progress in the ongoing push for the construction of quantum hardware with lower gate error rates, which will decrease the overhead cost of quantum error correction. This, in turn, will facilitate the faithful execution of more sophisticated quantum algorithms beyond the capabilities of modern classical computers, and help materialize the scientific and technological promises of the nascent second quantum revolution.

## Methods

### Data sets generation

In our numerical experiments, we adopted, both for input states and measurement operators, the set of the rank-1 projector into the eigenstates of the Pauli matrices:

9 $${M}_{0}={p}_{z} | 0 \rangle \, \langle 0 |,\,\,\,{M}_{1}={p}_{z} | 1 \rangle \, \langle 1 |,$$

10 $${M}_{2}={p}_{x} |+\rangle \, \langle+|,\,\,\,{M}_{3}={p}_{x} | -\rangle \,\langle - |,$$

11 $${M}_{4}={p}_{y} |+i \rangle \, \langle+i |,\,\,\,{M}_{5}={p}_{y} | -i \rangle \, \langle -i | $$

We assume throughout equal probabilities *p*_*x*_ = *p*_*y*_ = *p*_*z*_ = 1/3. The full set for the *N*-qubit system is obtained from the tensor product of the operators single-qubit operators

12 $${{{{{{{{\boldsymbol{M}}}}}}}}}_{{{{{{{{\boldsymbol{\alpha }}}}}}}}}={M}_{{\alpha }_{1}}\otimes {M}_{{\alpha }_{2}}\otimes \cdots \otimes {M}_{{\alpha }_{N}},$$

and it is specified by a string ***α*** = (*α*_1_, …, *α*_*N*_), with *α*_*j*_ = 0, …, 5. The input states are simple product states ${{{{{{{{\boldsymbol{\rho }}}}}}}}}_{{{{{{{{\boldsymbol{\alpha }}}}}}}}}={t}_{{{{{{{{\boldsymbol{\alpha }}}}}}}}}^{-1}{{{{{{{{\boldsymbol{M}}}}}}}}}_{{{{{{{{\boldsymbol{\alpha }}}}}}}}}$ with proper normalization ${t}_{{{{{{{{\boldsymbol{\alpha }}}}}}}}}={{{{{{{\rm{Tr}}}}}}}}\,{{{{{{{{\boldsymbol{M}}}}}}}}}_{{{{{{{{\boldsymbol{\alpha }}}}}}}}}={\prod }_{j}{{{{{{{\rm{Tr}}}}}}}}\,{M}_{{\alpha }_{j}}$. The measurement operators ***M***_***β***_ are defined analogously, and identified by a string ***β*** = (*β*_1_, …, *β*_*N*_).

We now provide the step-by-step procedure used to generate the training data for the case of the unitary quantum circuits. Even though the operators we implement are rank-1, we give a description for a more general case of an IC positive operator valued measures (POVM) ***M*** beyond the standard projective measurements. For a given circuit architecture, containing a set of single-qubit and two-qubit gates, we first contract each gate together to obtain the MPO corresponding to the full circuit unitary ***U***. After each application of a two-qubit gate, we restore the tensor network into an MPO structure by means of singular value decomposition. During this step, we only discard zero singular values, which implies that there is no approximation in the unitary MPO, and that the bond dimension *χ*_*U*_ generally grows exponentially with the depth of the circuit.

**Fig. 6:**
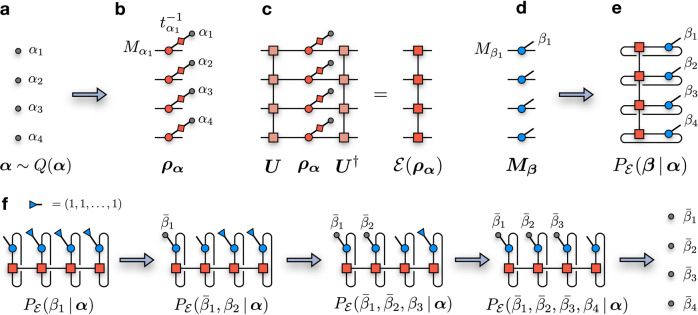
Generation of one training data sample. **a** First, we sample a random input POVM state ***α*** = (*α*_1_, *α*_2_, …, *α*_*N*_) from a reference prior distribution *Q*(***α***). **b** The string ***α*** specifies an input product state ${{{{{{{{\boldsymbol{\rho }}}}}}}}}_{{{{{{{{\boldsymbol{\alpha }}}}}}}}}={t}_{{{{{{{{\boldsymbol{\alpha }}}}}}}}}^{-1}{{{{{{{{\boldsymbol{M}}}}}}}}}_{{{{{{{{\boldsymbol{\alpha }}}}}}}}}$ to the channel. **c** The output state of the channel is obtained by contracting the input state with the circuit MPO ***U***, resulting into a new MPO ${{{{{{{\mathcal{E}}}}}}}}({{{{{{{{\boldsymbol{\rho }}}}}}}}}_{{{{{{{{\boldsymbol{\alpha }}}}}}}}})$. **d** The measurement POVM ***M***_***β***_. **e** The process probability distribution ${P}_{{{{{{{{\mathcal{E}}}}}}}}}({{{{{{{\boldsymbol{\beta }}}}}}}}|{{{{{{{\boldsymbol{\alpha }}}}}}}})={{{{{{{{\rm{Tr}}}}}}}}}_{{{{{{{{\boldsymbol{\tau }}}}}}}}}[{{{{{{{{\boldsymbol{M}}}}}}}}}_{{{{{{{{\boldsymbol{\beta }}}}}}}}}{{{{{{{\mathcal{E}}}}}}}}({{{{{{{\boldsymbol{{\rho }}}}}}}_{{{{{{{{\boldsymbol{\alpha }}}}}}}}}}})]$. **f** Sampling scheme to obtain a single measurement outcome ***β*** from ${P}_{{{{{{{{\mathcal{E}}}}}}}}}({{{{{{{\boldsymbol{\beta }}}}}}}}|{{{{{{{\boldsymbol{\alpha }}}}}}}})$. By tracing the indices *β*_2_, …, *β*_*N*_ (i.e., contracting with a vector [1, …, 1]), the resulting tensor network with one open index is the probability ${P}_{{{{{{{{\mathcal{E}}}}}}}}}({\beta }_{1}\,|\,{{{{{{{\boldsymbol{\alpha }}}}}}}})$, which can be sampled to generate a measurement outcome ${\bar{\beta }}_{1}$. By sweeping left to right, this procedure is repeated for each qubits, generating an outcome $\bar{{{{{{{{\boldsymbol{\beta }}}}}}}}}$ from the correct probability distribution ${P}_{{{{{{{{\mathcal{E}}}}}}}}}({{{{{{{\boldsymbol{\beta }}}}}}}}|{{{{{{{\boldsymbol{\alpha }}}}}}}})$. The final result of this procedure is one single training sample (***α***, ***β***). The data set are generated by repeating these steps consecutively.

Next, we fix a uniform prior distribution *Q*(***α***) = *K*^−*N*^ for the input states, where *K* is the cardinality of the single-qubit POVM (e.g., *K* = 6 for the Pauli projectors). The POVM string ***α*** is randomly sampled from *Q*(***α***) (Fig. 6a), which defines a specific input state (Fig. 6b)

13 $${{{{{{{{\boldsymbol{\rho }}}}}}}}}_{{{{{{{{\boldsymbol{\alpha }}}}}}}}}=\frac{{{{{{{{{\boldsymbol{M}}}}}}}}}_{{{{{{{{\boldsymbol{\alpha }}}}}}}}}}{{t}_{{{{{{{{\boldsymbol{\alpha }}}}}}}}}}=\frac{{M}_{{\alpha }_{1}}}{{t}_{{\alpha }_{1}}}\otimes \frac{{M}_{{\alpha }_{2}}}{{t}_{{\alpha }_{2}}}\otimes \cdots \otimes \frac{{M}_{{\alpha }_{N}}}{{t}_{{\alpha }_{N}}}$$

For the set of Pauli eigenstates projectors, this translates into applying one layer of single-qubit gates, according to the string ***α***. The output state of the channel is then estimated by contracting ***ρ***_***α***_ with the circuit MPO ***U***, ${{{{{{{\mathcal{E}}}}}}}}({{{{{{{{\boldsymbol{\rho }}}}}}}}}_{{{{{{{{\boldsymbol{\alpha }}}}}}}}})={{{{{{{\boldsymbol{U}}}}}}}}{{{{{{{{\boldsymbol{\rho }}}}}}}}}_{{{{{{{{\boldsymbol{\alpha }}}}}}}}}{{{{{{{{\boldsymbol{U}}}}}}}}}^{{{{\dagger}}} }$ (Fig. 6c). The output state ${{{{{{{\mathcal{E}}}}}}}}({{{{{{{{\boldsymbol{\rho }}}}}}}}}_{{{{{{{{\boldsymbol{\alpha }}}}}}}}})$ is itself an MPO describing a properly normalized density operator.

Given the output state and the measurement operator ***M***_***β***_ (Fig. 6d), the process probability ${P}_{{{{{{{{\mathcal{E}}}}}}}}}({{{{{{{\boldsymbol{\beta }}}}}}}}|{{{{{{{\boldsymbol{\alpha }}}}}}}})$ is obtained by contracting (and tracing) these two objects together (Fig. 6e). This probability can then be exactly sampled using the chain rule of probabilities^37,62^. The measurement probability for qubit 1 is computed as

14 $$p({\beta }_{1})=\mathop{\sum}\limits_{{\beta }_{2},{\beta }_{3},\ldots,{\beta }_{N}}p({\beta }_{1},{\beta }_{2},{\beta }_{3},\ldots,{\beta }_{N})$$

where we introduced the short-hand notation $p({{{{{{{\boldsymbol{\beta }}}}}}}})={P}_{{{{{{{{\mathcal{E}}}}}}}}}({{{{{{{\boldsymbol{\beta }}}}}}}}|{{{{{{{\boldsymbol{\alpha }}}}}}}})$. The probability *p*(*β*_1_) is calculated by tracing out each local POVM subspace via a contraction of the tensor network for ${P}_{{{{{{{{\mathcal{E}}}}}}}}}({{{{{{{\boldsymbol{\beta }}}}}}}}|{{{{{{{\boldsymbol{\alpha }}}}}}}})$ with constant vectors (1_1_, 1_2_, …, 1_*K*_) (blue triangles) at each site *j* = 2, …, *N* (Fig. 6f). Once known, the distribution can be sampled to generate measurement outcome ${\bar{\beta }}_{1} \sim P({\beta }_{1})$. Next, the probability distribution $p({\beta }_{2}|{\bar{\beta }}_{1})$ for the second qubit, conditional on the measurement of the first qubit, is calculated as the ratio between $p({\bar{\beta }}_{1},{\beta }_{2})$ (shown in the second network of Fig. 6f) and $p({\bar{\beta }}_{1})$. By repeating this procedure, one obtains a final configuration $\bar{{{{{{{{\boldsymbol{\beta }}}}}}}}}$ sampled from the correct probability distribution $p({{{{{{{\boldsymbol{\beta }}}}}}}})={P}_{{{{{{{{\mathcal{E}}}}}}}}}({{{{{{{\boldsymbol{\beta }}}}}}}}|{{{{{{{\boldsymbol{\alpha }}}}}}}})$. Importantly, each *N*-qubit measurement outcome is completely uncorrelated from any other.

For the noisy quantum channels studied in the paper, since there are only *N* = 5 qubits, we perform a direct simulation of the channel to obtain the full Choi matrix. The training data is obtained directly from the Choi matrix, using input states and measurement operators identical to the ones described above.

### Trace-preserving regularization

In general, the LPDO representation does not enforce the TP condition on the corresponding quantum channel, i.e., ${{{{{{{{\rm{Tr}}}}}}}}}_{{{{{{{{\boldsymbol{\tau }}}}}}}}}\,{{{{{{{{\boldsymbol{\Lambda }}}}}}}}}_{{{{{{{{\boldsymbol{\vartheta }}}}}}}}}\,\ne \,{{\Bbb{1}}}_{{{{{{{{\boldsymbol{\sigma }}}}}}}}}$. However, this condition can be easily added to the cost function as a *regularization term*, which biases the optimization to yield a set of optimal parameters ***ϑ*** that minimizes the negative log-likelihood, while also minimizing the *distance* between ${{{{{{{{\rm{Tr}}}}}}}}}_{{{{{{{{\boldsymbol{\tau }}}}}}}}}\,{{{{{{{{\boldsymbol{\Lambda }}}}}}}}}_{{{{{{{{\boldsymbol{\vartheta }}}}}}}}}$ and ${{\Bbb{1}}}_{{{{{{{{\boldsymbol{\sigma }}}}}}}}}$. As a distance measure, we choose the Frobenius norm of the difference ${{{{{{{{\mathbf{\Delta }}}}}}}}}_{{{{{{{{\boldsymbol{\vartheta }}}}}}}}}={{{{{{{{\rm{Tr}}}}}}}}}_{{{{{{{{\boldsymbol{\tau }}}}}}}}}{{{{{{{{\boldsymbol{\Lambda }}}}}}}}}_{{{{{{{{\boldsymbol{\vartheta }}}}}}}}}-{{\Bbb{1}}}_{{{{{{{{\boldsymbol{\sigma }}}}}}}}}$:

15 $$\parallel {{{{{{{{\mathbf{\Delta }}}}}}}}}_{{{{{{{{\boldsymbol{\vartheta }}}}}}}}}{\parallel }_{F}=\sqrt{{{{{{{{{\rm{Tr}}}}}}}}}_{{{{{{{{\boldsymbol{\sigma }}}}}}}}}\left({{{{{{{{\mathbf{\Delta }}}}}}}}}_{{{{{{{{\boldsymbol{\vartheta }}}}}}}}}{{{{{{{{\mathbf{\Delta }}}}}}}}}_{{{{{{{{\boldsymbol{\vartheta }}}}}}}}}^{{{{\dagger}}} }\right)}.$$

**Fig. 7:**
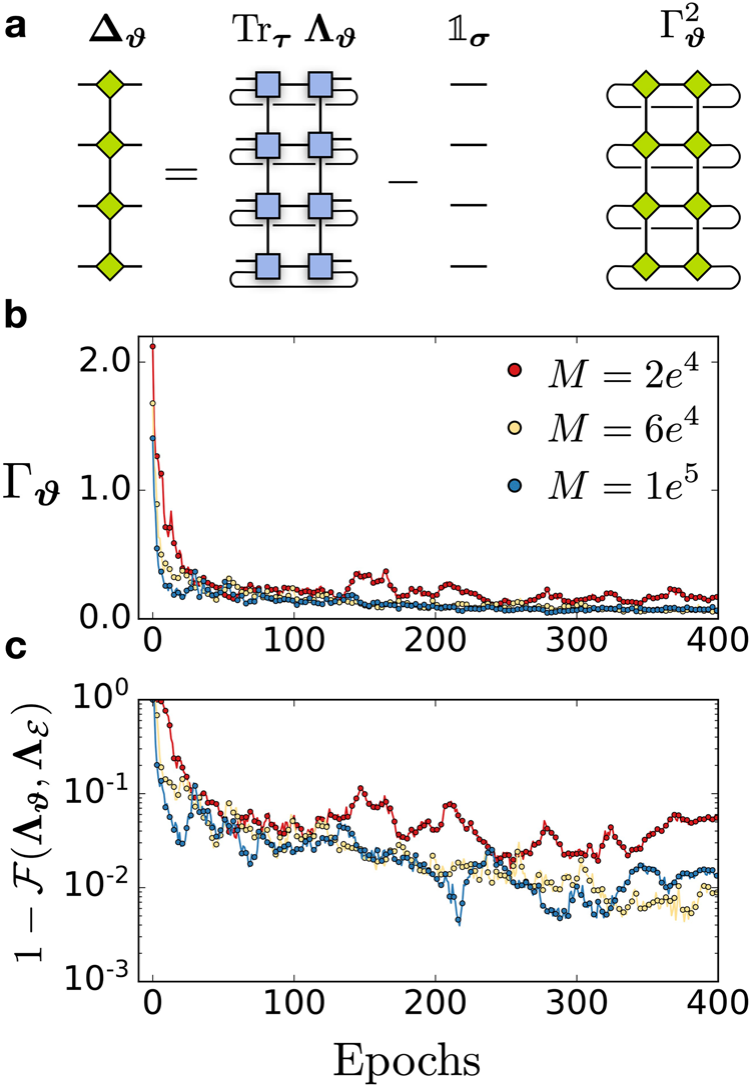
Trace-preserving regularization. **a** Tensor network for **Δ**_***ϑ***_, obtained by subtracting the identity MPO (with bond dimension 1) to the (properly normalized) LPDO **Λ**_***ϑ***_, and tensor contraction required to compute Γ_***ϑ***_. We show the measurement of the regularization Γ_***ϑ***_ (**b**) and the reconstruction infidelity (**c**) during the training for a one-dimension random quantum circuit with *N* = 10 qubits and depth *D* = 2, for different number of the total data set size *M*.

The tensor network for **Δ**_***ϑ***_ can be easily computed by performing an MPO subtraction^63^, which in this case it increases the bond dimension of **Λ**_***ϑ***_ by 1 (Fig. 7a). The regularization term is then

16 $${{{\Gamma }}}_{{{{{{{{\boldsymbol{\vartheta }}}}}}}}}=\sqrt{{d}^{-N}}\sqrt{{{{{{{{{\rm{Tr}}}}}}}}}_{{{{{{{{\boldsymbol{\sigma }}}}}}}}}\left({{{{{{{{\mathbf{\Delta }}}}}}}}}_{{{{{{{{\boldsymbol{\vartheta }}}}}}}}}{{{{{{{{\mathbf{\Delta }}}}}}}}}_{{{{{{{{\boldsymbol{\vartheta }}}}}}}}}^{{{{\dagger}}} }\right)},$$

where we introduced a normalization pre-factor $\sqrt{{d}^{-N}}$. This leads to the final cost function

17 $${{{{{{{\mathcal{C}}}}}}}}({{{{{{{\boldsymbol{\vartheta }}}}}}}})=\log {Z}_{{{{{{{{\boldsymbol{\vartheta }}}}}}}}}-\left\langle \right.\log {\widetilde{P}}_{{{{{{{{\boldsymbol{\vartheta }}}}}}}}}({{{{{{{\boldsymbol{\beta }}}}}}}}\,|\,{{{{{{{\boldsymbol{\alpha }}}}}}}}){\rangle }_{{{{{{{{\mathcal{D}}}}}}}}}+\kappa {{{\Gamma }}}_{{{{{{{{\boldsymbol{\vartheta }}}}}}}}},$$

where *κ* is an additional hyper-parameter.

We show the measurement of the regularization term Γ_***ϑ***_ (Fig. 7b) at each training iteration for the reconstruction of one-dimensional random quantum circuits of different depths. By comparing these curves with the reconstruction infidelities (Fig. 7c), one can clearly see the correlation between the accuracy of the reconstruction and the amount of violation of the TP condition.

### Overfitting and model selection

The goal of training the LPDO using unsupervised learning is to efficiently extract the relevant structure and features characterizing the unknown channel from a limited set of measurements. In other words, the model needs to be able to *generalize* beyond the measurements provided for its training. If the number of samples in the data set ${{{{{{{\mathcal{D}}}}}}}}$ is too low, it is likely that the LPDO training leads to *overfitting*, i.e., the LPDO learns features present in the data that are not representative of the unknown channel, but only stems from the limited number of training samples.

A strategy to monitor the overfitting, routinely used in the training of deep neural networks, is to divide the data set into two sub-sets: a training data set ${{{{{{{{\mathcal{D}}}}}}}}}_{T}$ and a validation data set ${{{{{{{{\mathcal{D}}}}}}}}}_{V}$. Here, we do so using a 80%/20% split ratio. The training data set ${{{{{{{{\mathcal{D}}}}}}}}}_{T}$ is used for the learning procedure, i.e., the calculation of the gradients used to update the model. During training, we compute the training loss (i.e., the average of the cost function on the training data set)

18 $${{{{{{{{\mathcal{L}}}}}}}}}_{T}({{{{{{{\boldsymbol{\vartheta }}}}}}}})=-\frac{1}{|{{{{{{{{\mathcal{D}}}}}}}}}_{T}|}\mathop{\sum}\limits_{({{{{{{{\boldsymbol{\alpha }}}}}}}},{{{{{{{\boldsymbol{\beta }}}}}}}})\in {{{{{{{{\mathcal{D}}}}}}}}}_{T}}\log {P}_{{{{{{{{\boldsymbol{\vartheta }}}}}}}}}({{{{{{{\boldsymbol{\beta }}}}}}}}|{{{{{{{\boldsymbol{\alpha }}}}}}}}),$$

which signals whether the model is actively learning (i.e., a decreasing ${{{{{{{{\mathcal{L}}}}}}}}}_{T}({{{{{{{\boldsymbol{\vartheta }}}}}}}})$). At the same time, we also compute the validation loss on the held-out data

19 $${{{{{{{{\mathcal{L}}}}}}}}}_{V}({{{{{{{\boldsymbol{\vartheta }}}}}}}})=-\frac{1}{|{{{{{{{{\mathcal{D}}}}}}}}}_{V}|}\mathop{\sum}\limits_{({{{{{{{\boldsymbol{\alpha }}}}}}}},{{{{{{{\boldsymbol{\beta }}}}}}}})\in {{{{{{{{\mathcal{D}}}}}}}}}_{V}}\log {P}_{{{{{{{{\boldsymbol{\vartheta }}}}}}}}}({{{{{{{\boldsymbol{\beta }}}}}}}}|{{{{{{{\boldsymbol{\alpha }}}}}}}}).$$

Here, $|{{{{{{{{\mathcal{D}}}}}}}}}_{T}|$ and $|{{{{{{{{\mathcal{D}}}}}}}}}_{V}|$ are the size of the training and validation data sets, respectively.

Generally, in the early stage of the training, the validation loss decreases hand-in-hand with the training loss. However, if the model starts to overfit spurious features in the training data, the validation loss will invert its trend and start increasing, an indication that more training data is needed. We stress that both of these measurements are available in a practical experimental setting, since no information about the channel is being used.

The validation loss ${{{{{{{{\mathcal{L}}}}}}}}}_{V}({{{{{{{\boldsymbol{\vartheta }}}}}}}})$ is also a useful metric to perform the model selection, i.e., to pick a specific set of parameters ***ϑ***^(*t*)^ at epoch *t* to be considered the optimal solution of the optimization problem. In our numerical simulations, we select the optimal parameters as the ones at the training epochs *t* where the measurement of the validation loss returned its lowest value. This is also a model selection procedure that can be used in an experimental setting.

### Specifics of the numerical experiments

In this final section, we provide details on the numerical experiments presented in the main text. In all cases, the LPDO tensors $\{{\widetilde{A}}_{j}\}$ are initialized randomly, with each tensor component set to

20 $${[{\widetilde{A}}_{j}]}_{{\mu }_{j-1},{\nu }_{j},{\mu }_{j}}^{{\tau }_{j},{\sigma }_{j}}={a}_{r}+i{a}_{i}$$

where *a*_*r*_ and *a*_*i*_ are drawn from a uniform distribution centered around zero with width 0.2. We compute the gradients on batches of data containing *M*_*B*_ = 800 samples. Once the gradients are collected, we update the LPDO tensors using the Adam optimization with parameters *η* = 0.005, *ξ*_1_ = 0.9, *ξ*_2_ = 0.999, and *ϵ* = 10^−7^.

*Figure 2*. The first set of quantum channels investigated are unitary quantum circuits containing one layer of single-qubit gates. We study two types of circuits, containing either Hadamard gates

21 $$H=\frac{1}{\sqrt{2}}\left(\begin{array}{ll}1&1\\ 1&-1\end{array}\right),$$

or random single-qubit rotations

22 $$R(\theta,\phi,\lambda )=\left(\begin{array}{ll}\cos \frac{\theta }{2}&-{e}^{i\lambda }\sin \frac{\theta }{2}\\ {e}^{i\phi }\sin \frac{\theta }{2}&{e}^{i(\phi+\lambda )}\cos \frac{\theta }{2}\end{array}\right).$$

To obtain the sample complexity curves shows in Fig. 2b, we perform the reconstruction for an increasing number *N* of qubits. For each *N*, we start using a small data set size *M*, and increase it with a fixed size-step until the threshold *ε* = 0.025 in infidelity is met. The result is a value M* with an error bar given by the size-step.

We repeat the same scaling study for quantum circuits containing *D* layers of controlled-NOT (CX) gates

23 $${{{{{{{\rm{CX}}}}}}}}=\left(\begin{array}{llll}1&0&0&0\\ 0&1&0&0\\ 0&0&0&1\\ 0&0&1&0\end{array}\right).$$

For a quantum circuit with depth *D*, the odd and even layers apply two-qubit gates with the control qubit having odd and even qubit-index, respectively. Here, the bond dimension of the LPDO Choi matrix is set to the bond dimension of the circuit MPO.

*Figure 3*. We reconstruct random quantum circuits in both one and two dimensions. In both cases, each layer of the quantum circuit consists of one layer with *N* single-qubit random rotations *R*(*θ*, *ϕ*, *λ*) (defined above) and one layer of CX gates. In the one-dimensional geometry, the CX gates alternates as in the previous case. For the two-dimensional quantum circuit, they are applied according to the color scheme shown in Fig. 3b. For the simulation of the quantum circuit and the data generation, the circuit MPO has a “snake-shape” as per usual in MPS simulations of two-dimensional geometries. After applying the CX gates, the circuit tensor network is restored into a local form by means of singular value decomposition, where only zero singular values are discarded. This means that the representation of the target quantum circuit is exact.

We first set of the bond dimension of the LPDO Choi matrix equal to the bond dimension of the circuit MPO, and set the Kraus dimension to *χ*_*ν*_ = 1. All the data shown in Fig. 3 has been collected under this condition. However, additional simulations have also been performed using larger values of the LPDO bond dimension, obtaining comparable results. During the training, we monitor the training loss, the validation loss, the TP regularizer, and the reconstruction fidelity. We use cross-validation on the held-out data set ${{{{{{{{\mathcal{D}}}}}}}}}_{V}$ to select the best models for each circuit configuration and for each data set size *M*. The curves in Fig. 3e, f show the reconstruction infidelities of these selected models.

*Figure 4*. Finally, we reconstruct a noisy quantum channel. We consider the X-stabilizer measurement in the surface code, where the parity-check between four data qubits is measured using an additional (measurement) qubit with the quantum circuit shown in Fig. 4a. The circuit contains two Hadamard gates and four CX gates. We apply an amplitude-damping channel, characterized by the Kraus operators

24 $${K}_{0}=\left|0\right\rangle \left\langle 0\right |+\sqrt{1-\gamma }\left|1\right\rangle \left\langle 1\right|$$

25 $${K}_{1}=\sqrt{\gamma }\left|0\right\rangle \left\langle 1\right|$$

where *γ* is the decay probability. The channel is applied to each quantum gate in the circuit, where for the two-qubit gates the channel is just the tensor product of the single-qubit channel shown above.

We now relax any prior information on both the quantum circuit and the noise channel. We perform the reconstruction by varying the bond dimension *χ*_*μ*_ and the Kraus dimension *χ*_*ν*_ of the LPDO. The only setting where convergence in the training metrics is found already for *χ*_*ν*_ = 0 is the noiseless channel *γ* = 0, as expected. Nonetheless, even by increasing *χ*_*ν*_, the noiseless channel is still properly reconstructed. This can be seen in Fig. 4b, where the purity of the reconstruction LPDO Choi matrix for *γ* = 0 and *χ*_*ν*_ = 6 reaches the correct value of ${{{{{{{\rm{Tr}}}}}}}}{{{{{{{{\boldsymbol{\Lambda }}}}}}}}}_{{{{{{{{\boldsymbol{\vartheta }}}}}}}}}\,\approx \,1$. The infidelity curves are shown for a fixed data set size of *M* = 5 × 10^5^.

## Supplementary information

Supplementary Information

## Data Availability

The data sets generated during and/or analyzed during the current study are available from the corresponding author on request.

## Code availability

The software used in this work has been re-based into the Julia package PastaQ^65^.
