# Supplementary material for: Quantum process tomography with unsupervised learning and tensor networks
Source: Nat Commun. 2023 May 19;14:2858. doi: 10.1038/s41467-023-38332-9 (PMC10199030; doi:10.1038/s41467-023-38332-9)
Supplement: Supplementary file 1 — Supplementary Information [file 41467_2023_38332_MOESM1_ESM.pdf]

Quantum process tomography with unsupervised learning and tensor networks: supplementary information

Giacomo Torlai,[1,2,*] Christopher J. Wood,[3] Atithi Acharya,[2,4]
Giuseppe Carleo,[2,5] Juan Carrasquilla,[6] and Leandro Aolita[7,8]

[1] *AWS Center for Quantum Computing, Pasadena, CA, USA*
[2] *Center for Computational Quantum Physics, Flatiron Institute, New York, NY 10010, USA*
[3] *IBM T.J. Watson Research Center, Yorktown Heights, NY 10598, USA*
[4] *Physics and Astronomy Department, Rutgers University, Piscataway, NJ 08854, USA*
[5] *Institute of Physics, École Polytechnique Fédérale de Lausanne, CH-1015 Lausanne, Switzerland*
[6] *Vector Institute, MaRS Centre, Toronto, Ontario, M5G 1M1, Canada*
[7] *Quantum Research Centre, Technology Innovation Institute, Abu Dhabi, UAE*
[8] *Instituto de Física, Federal University of Rio de Janeiro, 21941-972, P. O. Box 68528, Rio de Janeiro, Brazil*

Tensor networks for Choi matrices

Due to the intrinsic exponential scaling of exact classical representations of quantum states and operators, a direct estimation of the Choi matrix given the knowledge of the channel is limited to a very small number of qubits. One may wonder if, for a subset of quantum channels that admit an efficient representation (e.g. low-depth quantum circuits), the Choi matrix can be also computed efficiently. To this end, we adopt a tensor network representation of the Choi matrix, which for a N-qubit quantum channel is a rank-$4N$ tensor, with $2N$ input indices and $2N$ output indices [1]. In its *canonical form*, the input and output indices are placed on the upper and lower half of the full tensor respectively.

We simplify the discussion and assume that the quantum channel is noiseless, i.e. it implements a unitary evolution $\mathcal{E}:\ \boldsymbol{\rho} \longrightarrow \boldsymbol{U}\boldsymbol{\rho}\boldsymbol{U}^\dagger$, where $\boldsymbol{U}$ corresponds to a quantum circuits compiled into one- and two-qubit gates. Depending on the type of gates, the geometry of the circuit and its depth, the unitary $\boldsymbol{U}$ may admit an efficient representation as a matrix product operator (MPO)

$$\boldsymbol{U}_{\boldsymbol{\sigma}\boldsymbol{\sigma}'} = \sum_{\{\boldsymbol{\mu}\}} \prod_{j=1}^{N} [B_j]^{\sigma_j,\sigma_j'}_{\mu_{j-1},\mu_j}\ , \tag{1}$$

where $\boldsymbol{U}_{\boldsymbol{\sigma}\boldsymbol{\sigma}'} = \langle\boldsymbol{\sigma}|\boldsymbol{U}|\boldsymbol{\sigma}'\rangle$. Each B_j is a rank-4 tensor with physical indices (σ_j,σ_j') and *bond indices* (μ_{j-1},μ_j) (Supplementary Fig. 1a). The *bond dimension* of the MPO is defined as the maximum dimension of any bond index $\chi_U = \max_j\{\chi_{\mu_j}|\chi_{\mu_j} = \dim[\mu_j]\}$, and it represents the measure of complexity of the MPO representation of the unitary $\boldsymbol{U}$.

The straightforward way to obtain a tensor network representation of the Choi matrix (for a unitary circuit) is to simply apply the circuit MPO to the (unnormalized) density operator $\boldsymbol{\Phi} = |\Phi^+\rangle\langle\Phi^+|^{\otimes N}$ for the N unnormalized Bell pairs, each described by a matrix product state (MPS) with bond dimension $\chi_{\Phi^+} = 2$ (Supplementary Fig. 1b). In doing so, there is freedom in how the contractions between the circuit MPO and Bell state MPS is done, stemming from different arrangements of the indices of $\boldsymbol{\Phi}$. If one were to pursue the Choi matrix in its canonical form, the MPS indices should to be properly swapped before the contraction with the MPO (Supplementary Fig. 1c). This however results in a very inefficient tensor network representation, as it brings the tensor product of N Bell pairs into a $2N$-qubit maximally entangled state, which saturates the bond dimension of the MPS to $\chi_{\boldsymbol{\Phi}} = 2^N$.

In practice, there is no particular reason to keep the Choi matrix in its canonical form, and an efficient representation is instead obtained as follows. First, we contract the circuit MPO with the physical indices of each Bell pair MPS (leaving out the ancilla qubits). Because the Bell state is equivalent to the vectorization of the identity matrix, the contraction between the circuit MPO and the full MPS simply returns the MPO itself. The inner (ancillary) indices can then be folded back into each local MPO tensor (Supplementary Fig. 1d). The result of this operation, i.e. the Choi matrix, is a rank-1 density matrix written in terms of an MPS with physical dimension d^2 and bond dimension χ_U. Thus, the Choi matrix can be obtained efficiently as long as the MPO bond dimension is sufficiently low. Note that this operation is also called *unravelling* in the context of column-vectorization of dense matrices [1]. In the case of a tensor product channel $\mathcal{E} = \mathcal{E}_1 \otimes \cdots \otimes \mathcal{E}_N$, the Choi matrix obtained in this form is the tensor product of the individual sub-system Choi matrices $\boldsymbol{\Lambda}_{\mathcal{E}} = \Lambda_{\mathcal{E}_1} \otimes \Lambda_{\mathcal{E}_2} \otimes \cdots \otimes \Lambda_{\mathcal{E}_N}$. This would not be the case if the one adopted the canonical ordering of the indices (Supplementary Fig. 1b), leading to $\boldsymbol{\Lambda}_{\mathcal{E}} = \boldsymbol{\Lambda}_{\mathcal{E}_1\otimes\mathcal{E}_2\otimes\cdots\otimes\mathcal{E}_N}$.

Tensor-network gradients

The parameters of the LPDO – the tensor components $\boldsymbol{\vartheta} = \{\widetilde{A}_j\}$ – are variationally optimized by minimizing

* Work done prior to Amazon; gttorlai@amazon.com

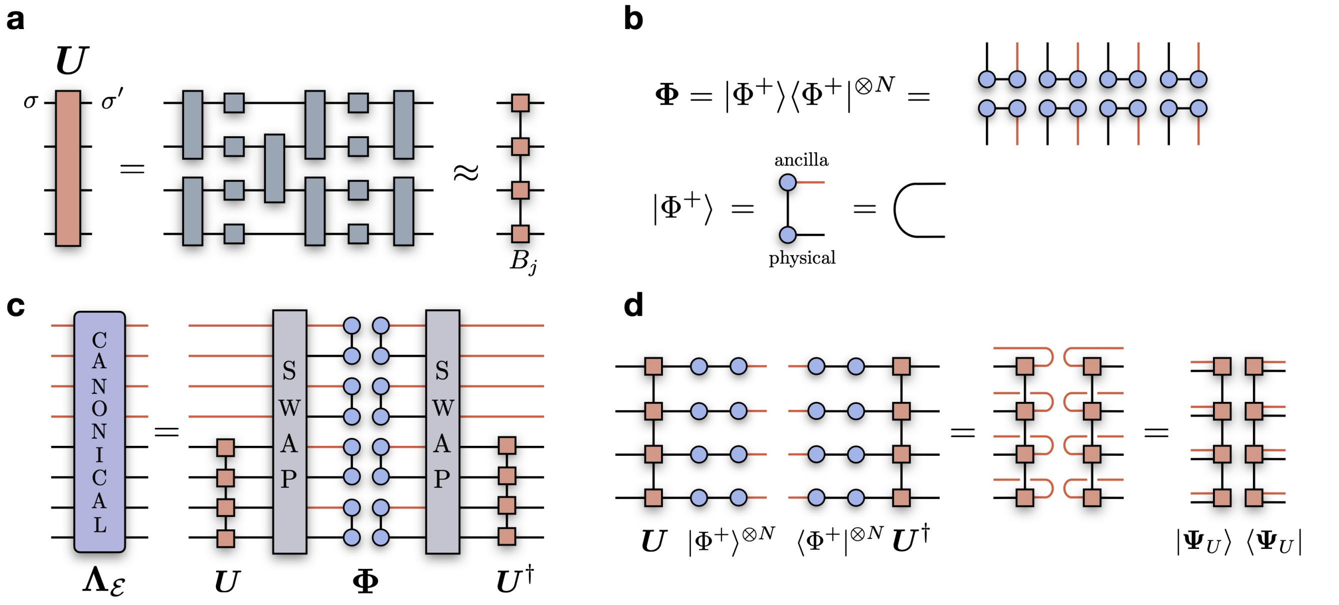

Supplementary Figure 1. Tensor network construction of the Choi matrix. (**a**) A unitary quantum operation $\boldsymbol{U}$ (with $N=4$ qubits), compiled into a set of single- and two-qubit quantum gates. By contracting all gates together, the unitary is approximated as an MPO with bond dimension χ_U. (**b**) The density operator $\boldsymbol{\Phi} = |\Phi^+\rangle\langle\Phi^+|^{\otimes N}$ for the tensor product of N unnormalized Bell pairs $|\Phi^+\rangle = |00\rangle + |11\rangle$. Each Bell pairs is an MPS with bond dimension $\chi_{\Phi^+} = 2$, corresponding to the vectorization of the identity matrix. (**c**) Canonical representation of the Choi matrix as a rank-$4N$ tensor. Within a one-dimensional tensor network representation, the canonical Choi matrix is obtained by first swapping the indices of the Bell pairs to bring all input (red) and output (black) indices together, and then contracting the resulting network with the unitary MPO on both sides. The dimension of the center bond after this operation is 2^N. (**d**) Efficient construction of the Choi matrix. Each Bell pair MPS is rearranged horizontally, corresponding to an identity matrix. The contraction with the unitary MPO is then trivial, and by folding the inner (input) indices back into each single MPO tensor, the resulting Choi matrix writes as a rank-1 density operator with physical dimension d^2.

the Kullbach-Leibler (KL) divergence

$$\mathcal{D}_{KL} = \sum_{\{\boldsymbol{\alpha}\}} Q(\boldsymbol{\alpha}) \sum_{\{\boldsymbol{\beta}\}} P_{\mathcal{E}}(\boldsymbol{\beta}\,|\,\boldsymbol{\alpha}) \log \frac{P_{\mathcal{E}}(\boldsymbol{\beta}\,|\,\boldsymbol{\alpha})}{P_{\boldsymbol{\vartheta}}(\boldsymbol{\beta}\,|\,\boldsymbol{\alpha})}\,. \qquad (2)$$

where $P_{\mathcal{E}}(\boldsymbol{\beta}\,|\,\boldsymbol{\alpha})$ is the process probability defined in Eq. (3) in the main text. The probability distribution $P_{\boldsymbol{\vartheta}}(\boldsymbol{\beta}\,|\,\boldsymbol{\alpha})$ associated to the process described by the LPDO is

$$\begin{aligned} P_{\boldsymbol{\vartheta}}(\boldsymbol{\beta}\,|\,\boldsymbol{\alpha}) &= \mathrm{Tr}_{\boldsymbol{\tau},\boldsymbol{\sigma}}\Big[(\boldsymbol{\rho}^T_{\boldsymbol{\alpha}} \otimes \boldsymbol{M}_{\boldsymbol{\beta}})\boldsymbol{\Lambda}_{\boldsymbol{\vartheta}}\Big] \\ &= Z^{-1}_{\boldsymbol{\vartheta}} d^N \mathrm{Tr}_{\boldsymbol{\tau},\boldsymbol{\sigma}}\Big[(\boldsymbol{\rho}^T_{\boldsymbol{\alpha}} \otimes \boldsymbol{M}_{\boldsymbol{\beta}})\widetilde{\boldsymbol{\Lambda}}_{\boldsymbol{\vartheta}}\Big] \\ &\equiv Z^{-1}_{\boldsymbol{\vartheta}} \widetilde{P}_{\boldsymbol{\vartheta}}(\boldsymbol{\beta}\,|\,\boldsymbol{\alpha})\,, \end{aligned} \qquad (3)$$

where $\boldsymbol{\Lambda}_{\boldsymbol{\vartheta}} = d^N Z^{-1}_{\boldsymbol{\vartheta}} \widetilde{\boldsymbol{\Lambda}}_{\boldsymbol{\vartheta}}$ is the properly normalized LPDO Choi matrix, and we defined the unnormalized LPDO probability distribution $\widetilde{P}_{\boldsymbol{\vartheta}}(\boldsymbol{\beta}\,|\,\boldsymbol{\alpha})$. By averaging Eq. (2) over the data set $\mathcal{D}$, we obtain the negative log-likelihood

$$\begin{aligned} \mathcal{C}(\boldsymbol{\vartheta}) &= -\frac{1}{M}\sum_{k=1}^{M} \log P_{\boldsymbol{\vartheta}}(\boldsymbol{\beta}_k\,|\,\boldsymbol{\alpha}_k) \\ &= \log Z_{\boldsymbol{\vartheta}} - \frac{1}{M}\sum_{k=1}^{M} \log \widetilde{P}_{\boldsymbol{\vartheta}}(\boldsymbol{\beta}_k\,|\,\boldsymbol{\alpha}_k) \\ &= \log Z_{\boldsymbol{\vartheta}} - \big\langle \log \widetilde{P}_{\boldsymbol{\vartheta}}(\boldsymbol{\beta}\,|\,\boldsymbol{\alpha})\big\rangle_{\mathcal{D}}\,, \end{aligned} \qquad (4)$$

where we omitted the constant entropy term of the target distribution.

Given the cost function $\mathcal{C}(\boldsymbol{\vartheta})$, the LPDO parameters are tuned according to the gradients

$$\begin{aligned} \mathcal{G}_{\boldsymbol{\vartheta}} &= \frac{\mathcal{C}(\boldsymbol{\vartheta})}{\partial\boldsymbol{\vartheta}} = \frac{\partial}{\partial\boldsymbol{\vartheta}} \log Z_{\boldsymbol{\vartheta}} - \frac{\partial}{\partial\boldsymbol{\vartheta}} \big\langle \log \widetilde{P}_{\boldsymbol{\vartheta}}(\boldsymbol{\beta}\,|\,\boldsymbol{\alpha})\big\rangle_{\mathcal{D}} \\ &= \frac{1}{Z_{\boldsymbol{\vartheta}}}\frac{\partial Z_{\boldsymbol{\vartheta}}}{\partial\boldsymbol{\vartheta}} - \Big\langle \frac{1}{\widetilde{P}_{\boldsymbol{\vartheta}}(\boldsymbol{\beta}\,|\,\boldsymbol{\alpha})} \frac{\partial \widetilde{P}_{\boldsymbol{\vartheta}}(\boldsymbol{\beta}\,|\,\boldsymbol{\alpha})}{\partial\boldsymbol{\vartheta}} \Big\rangle_{\mathcal{D}}\,. \end{aligned} \qquad (5)$$

Since, in general, the tensor components $\{\widetilde{A}_j\}$ are complex-valued, one should adopt the Wirtinger derivatives, and update each tensor $\widetilde{A}_j$ with the gradient taken with respect to its conjugate value $\widetilde{A}^*_j$.

The calculation of the gradients proceeds in two steps [2, 3]. First one evaluates the normalization $Z_{\boldsymbol{\vartheta}}$

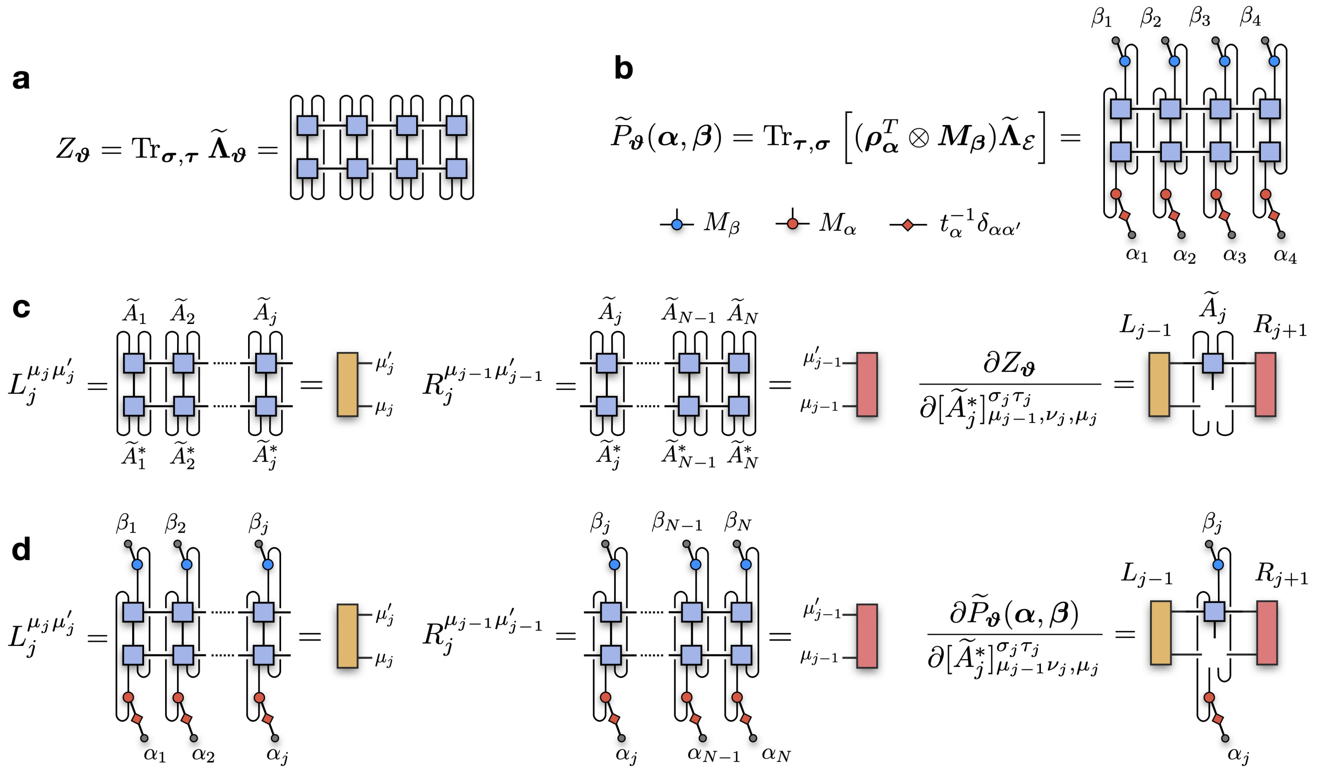

Supplementary Figure 2. Tensor-network optimization. (**a**) Tensor network contraction to evaluate the normalization $Z_{\boldsymbol{\vartheta}}$ of the LPDO. (**b**) Tensor network contraction to evaluate the unnormalized probability $\widetilde{P}_{\boldsymbol{\vartheta}}(\boldsymbol{\beta}\,|\,\boldsymbol{\alpha})$. (**c**) Calculation of the gradients of the normalization $\partial_{\boldsymbol{\vartheta}} Z_{\boldsymbol{\vartheta}}$. First, the left-environment tensors L_j ($j = 1,\dots,N-1$) are computed sequentially (and stored) by contracting the Kraus index and tracing the input/output index of the LPDO, from left to right. The same is repeated by contracting from right to left for the right-environment tensors R_j ($j = 2,\dots,N$). Then, the gradients of the normalization with respect to the conjugate tensor $\widetilde{A}^*_j$ are evaluated using with previously stored environment tensors. (**d**) Calculation of the gradients of the unnormalized probability $\partial_{\boldsymbol{\vartheta}}\widetilde{P}_{\boldsymbol{\vartheta}}(\boldsymbol{\alpha},\boldsymbol{\beta})$ for one given data point $(\boldsymbol{\alpha},\boldsymbol{\beta})$. As for the normalization, left and right environment tensors are calculated – where now the LPDO is contracted with input and output states corresponding to $\boldsymbol{\alpha}$ and $\boldsymbol{\beta}$ respectively – and subsequently used to evaluate the gradients with respect to each conjugate tensor $\widetilde{A}^*_j$.

with a trace over all the input/output indices of the LPDO (Supplementary Fig. 2a). The gradient of $Z_{\boldsymbol{\vartheta}}$ with respect to the component $\widetilde{A}^*_j$ corresponds to the tensor network used to compute $Z_{\boldsymbol{\vartheta}}$ with the tensor $\widetilde{A}^*_j$ removed from it. To reduce the number of tensor contractions required to compute the full set of gradients, one should first calculate and store the set of *environment tensors* $\{L_j\}$ and $\{R_j\}$ shown in Supplementary Fig. 2c, obtained in two sweeps over the LPDO respectively from left to right and from right to left. The gradients of the normalization with respect to each tensor $\widetilde{A}^*_j$ are then calculated with a third sweep as

$$\frac{\partial Z_{\boldsymbol{\vartheta}}}{\partial[\widetilde{A}^*_j]^{\sigma_j\tau_j}_{\mu_{j-1},\nu_j,\mu_j}} = \sum_{\mu'_j,\mu'_{j-1}} L_{j-1}^{\mu_{j-1},\mu'_{j-1}} [\widetilde{A}_j]^{\sigma_j\tau_j}_{\mu'_{j-1},\nu_j,\mu'_j} R_{j+1}^{\mu_j,\mu'_j}\,. \tag{6}$$

The second step repeats this procedure for the data-dependent term in the cost function. For each single data point $(\boldsymbol{\alpha},\boldsymbol{\beta})$, one computes the unnormalized probability $\widetilde{P}_{\boldsymbol{\vartheta}}(\boldsymbol{\beta}\,|\,\boldsymbol{\alpha})$ by contracting the LPDO with the corrersponding input state $\boldsymbol{\rho}_{\boldsymbol{\alpha}}$ and measurement operator $\boldsymbol{M}_{\boldsymbol{\beta}}$ (Supplementary Fig. 2b). One then sweeps through the LPDO to accumulate the left and right environment tensors, and compute the gradient of the unnormalized probability analogously to the normalization (Supplementary Fig. 2d). The final gradients are simply the average over all data samples.

Gradient updates

Once the gradients $\mathcal{G}_{\boldsymbol{\vartheta}}$ with respect to each tensor components are known, the LPDO is updated using gradient descent. In its simplest form, the parameters are changed according to

$$\boldsymbol{\vartheta} \longrightarrow \boldsymbol{\vartheta} - \eta\,\mathcal{G}_{\boldsymbol{\vartheta}}\,, \tag{7}$$

where η is the size of the update (i.e. the *learning rate*), and the gradients are computed over the full training

data set:

$$\mathcal{G}_{\boldsymbol{\vartheta}} = -\frac{1}{M}\sum_{k=1}^{M}\frac{\partial}{\partial\boldsymbol{\vartheta}}\log P_{\boldsymbol{\vartheta}}(\boldsymbol{\beta}_k \mid \boldsymbol{\alpha}_k)\,. \tag{8}$$

This type of gradient update results however into a very slow training, as the number of training samples M might be large. In practice, the gradients are computed on a *batch* of data containing $M_B \ll M$ training samples. One training *epoch* is then defined as a sweep of the full data set $\mathcal{D}$ (being reshuffled at its start), with the parameters $\boldsymbol{\vartheta}$ being updated M/M_B times. The advantage, aside a faster training, is that the fluctuations induced in the gradients due to the smaller number of samples M_B helps the optimization to escape local minima.

The other hyper-parameter of the optimization is the learning rate η. A major limitation of the vanilla gradient descent update shown above is that choosing the correct value of η in advance can be difficult. Further, the learning rate is identical for all parameters. This problem is resolved by using more advanced optimizations schemes. We specificallu use the Adam optimizer (from *Adaptive Moment Estimation*) [4], where each parameter ϑ_k is updated with an adaptive learning rate η_k. For the set of parameters $\boldsymbol{\vartheta}^{(t)}$ at a given epoch t, the Adam optimizer estimates moving averages of first and second moments of the gradients

$$\mathcal{M}_{1,k}^{(t)} = \xi_1\mathcal{M}_{1,k}^{(t-1)} + (1-\xi_1)\,\mathcal{G}_{\vartheta_k} \tag{9}$$
$$\mathcal{M}_{2,k}^{(t)} = \xi_2\mathcal{M}_{2,k}^{(t-1)} + (1-\xi_2)\,\mathcal{G}_{\vartheta_k}^2\,, \tag{10}$$

where ξ_1 and ξ_2 are hyper-parameters controlling the rate of decay of the moving averages. Each parameters is then updated as

$$\vartheta_k^{(t+1)} = \vartheta_k^{(t)} - \eta\frac{\widehat{\mathcal{M}}_{1,k}^{(t)}}{\sqrt{\widehat{\mathcal{M}}_{2,k}^{(t)}} + \epsilon}\,, \tag{11}$$

where ϵ is used to avoid numerical instabilities, and $\widehat{\mathcal{M}}_{i,k}^{(t)} = \mathcal{M}_{i,k}^{(t)}/(1-\xi_i)$ are used to correct the bias introduced by the zero-initialization $\mathcal{M}_{i,k}^{(t=0)} = 0$ [4].

[1] Christopher J. Wood, Jacob D. Biamonte, and David G. Cory, "Tensor networks and graphical calculus for open quantum systems," Quantum Information and Computation **15**, 0579–0811 (2015).

[2] Zhao-Yu Han, Jun Wang, Heng Fan, Lei Wang, and Pan Zhang, "Unsupervised generative modeling using matrix product states," Physical Review X **8**, 031012– (2018).

[3] Ivan Glasser, Ryan Sweke, Nicola Pancotti, Jens Eisert, and J. Ignacio Cirac, "Expressive power of tensor-network factorizations for probabilistic modeling, with applications from hidden Markov models to quantum machine learning," arXiv e-prints , arXiv:1907.03741 (2019), arXiv:1907.03741 [cs.LG].

[4] Diederik P. Kingma and Jimmy Ba, "Adam: A method for stochastic optimization," (2014), arXiv:1412.6980 [cs.LG]
